# Physical Insight on Mechanism of Photoinduced Charge Transfer in Multipolar Photoactive Molecules

**DOI:** 10.1038/s41598-018-28429-3

**Published:** 2018-07-04

**Authors:** Yuanzuo Li, Chaofan Sun, Peng Song, Fengcai Ma, Nawee Kungwan, Mengtao Sun

**Affiliations:** 1 College of Science, Northeast Forestry University, Harbin, 150040 Heilongjiang China; 2 Institute of Atomic and Molecular Physics, Jilin University, Changchun, 130012 China; 3 Department of Physics, Liaoning University, Shenyang, 110036 China; 4 School of Mathematics and Physics, Beijing Key Laboratory for Magneto-Photoelectrical Composite and Interface Science, University of Science and Technology Beijing, Beijing, 100083 China; 5 Department of Chemistry, Faculty of Science, Chiang Mai University, Chiang Mai, 50200 Thailand; 6 Center of Excellence in Materials Science and Technology, Chiang Mai University, Chiang Mai, 50200 Thailand

## Abstract

Two series of novel dyes were designed based on the multipolar structures of the red dye D35 and blue dye DB, by introducing the furan (F), benzene ring (B) and benzo[*c*]thiophene (BT) groups into the conjugated bridge of D35 in proper order and adjusting the position of diketopyrrolopyrrole(DPP) unit and the incorporation of fluorine in the conjugated bridge of DB, respectively. We performed the quantum chemistry calculation to investigate the ground state and excited properties in a direct correlation with the spectra properties and abilities of losing or accepting electron for the original and designed molecules. Furthermore, the absorption spectra characteristics in consideration of the aggregation of dyes on the TiO_2_ layer and intermolecular charge transfer rate of the dimers were calculated. The obtained results indicate that the larger intermolecular charge transfer rate leads to the poor photoelectrical properties of the dyes, and the designed dyes D35-3 and DB-2 would exhibit the best photoelectrical properties among the investigated dyes due to their lower energy gaps, widened absorption spectra and prominent charge transfer properties.

## Introduction

At present, energy generation mainly comes from the fossil fuels, for which nonrenewable and chemical properties make energy crisis and environmental pollution inevitable. To be a kind of pure renewable source, solar energy shows the advantages of unlimited reserves and no pollution, which is incomparable over other energy sources. As a device that converts sunlight into electrical energy, the solar cell has been paid more and more attention in recent decades^1–7^. Compared with the solar cells based on semiconductor silicon with the disadvantages of complex production process, high production cost and not large-scale application, etc., the dye-sensitized solar cells (DSSCs) have their advantages of low cost, environmental friendly, simple fabrication process and easy manufacture on a large scale^8^. In 1991, Grätzel *et al*.^9^ reported a kind of DSSC with the 7.1–7.9% of photoelectrical conversion efficiency (PCE) in simulated solar light under the condition of using the porous TiO_2_ film as the anode material and a monolayer of trimeric ruthenium complex as photo-sensitizer, which made a breakthrough in the DSSCs field.

In general, the basic structure of DSSC mainly consists of photo-anode, dye, electrolyte and counter electrode, in which the dye determining the ability to capture light and electron transfer plays a vital role in DSSCs. The photocurrent generated in DSSC requires the following steps^10^: (a) firstly, the dyes absorbing the sunlight are excited, with subsequent excited electron injection into TiO_2_; (b) the oxidized dyes are regenerated through obtaining electrons from the electrolyte; (c) the electrons in the TiO2 are transmitted to the photo-anode through the nanocrystalline film, and then transferred to the counter electrode through an external circuit; (d) the oxidized electrolyte is reduced through obtaining electrons from the counter electrode, thereby completing a cycle from sunlight to electrical energy. Nowadays, the dyes are mainly divided into ruthenium (Ru) dyes^11–13^, porphyrin dyes^14–16^ and metal-free dyes^17–20^, in which the metal-free dyes have the excellent characteristics such as lower manufacture cost, higher molar extinction coefficient and adjustable spectral properties in comparison with the metal-containing dyes. In recent years, researchers have undertaken extensive research on the application of metal-free dyes in the field of DSSCs, in which the breakthrough about the PCE of DSSCs up to 13% has been reported^21^. Hagberg *et al*.^22^ reported a various of chromophores (D29, D35 and D37) based on the triphenylamine (TPA) chromophore, in which solar cell based on D35 exhibited the highest PCE of 6.0% due to the large steric bulk originated from the four butoxy groups. Yao *et al*.^23^ synthesized a dye containing N-annulated indenoperylene with efficiency of 12.5% under normal irradiance without use of any coadsorbate. Based on phenothiazine with trilateral π-conjugation extensions, Iqbal and collaborators^24^ reported the dyes TLEP-1 and TLEP-2, where the TLEP-2 with the trilateral π-conjugation extensions had displayed a high short-circuit current density (*J*_*SC*_).

Although the considerable research works on enhancing the photoelectrical properties of dyes have been done in the experiment, it is still a challenge to understand the micro-mechanism. In recent decades, the theoretical calculation based on quantum chemical method has been considered as an effective way to reveal structure-activity relationship^25–30^. Mehmood *et al*.^31^ explored the benzene/thiophene sensitizers and the dye/TiO_2_ complexes by using Kohn-Sham density functional theory (DFT) and time-dependent density functional theory (TD-DFT), which indicated that D3 could be as the most suitable sensitizer owing to its most negative electron injection force (0.91 eV) and a larger light harvesting efficiency (0.95). Ferdowsi *et al*.^32^ studied the frontier molecular orbital (FMO) energy and optical absorption of four novel organic dyes containing phenoxazine as electron donor, thiophene and cyanovinylene linkers as the *π*-bridge, which indicated that D2–4 dyes could be suitable candidates as sensitizers. Feng *et al*.^33^ reported the electronic structures and aggregation properties of different phenothiazine system using DFT and Marcus theory to reveal the reason of the low dye regeneration.

**Figure 1:**
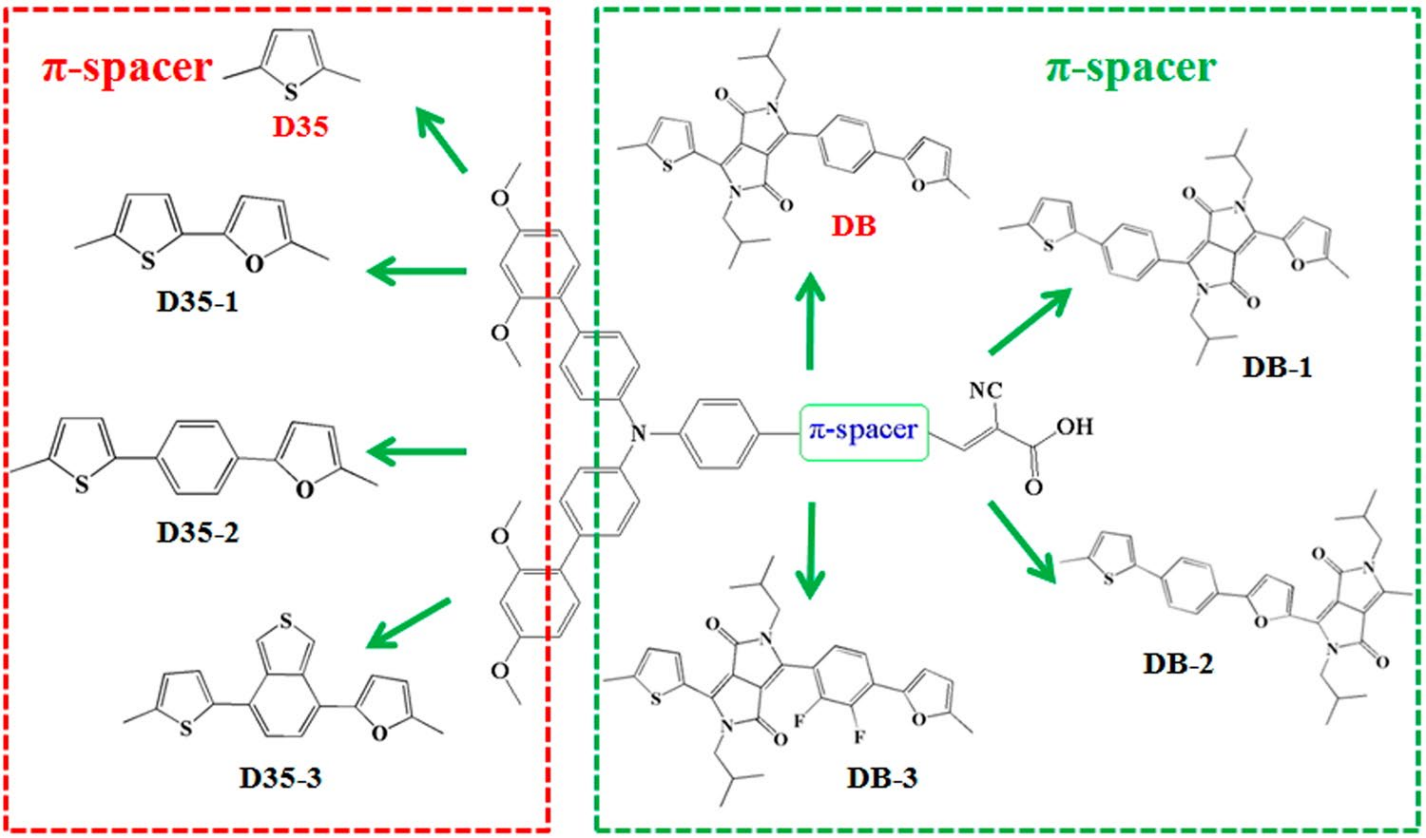
Modification strategy for the π-conjugated bridge of original dyes D35 and DB.

Recently, Hao *et al*.^34^ designed and synthesized a novel blue D-π-A dye (Dyenamo Blue, DB) with a diketopyrrolopyrrole (DPP)-core, which was designed on the basis of the structure of red dye D35, and the introduction of the DPP structure into the dye DB exhibited a promising performance of 7.3% with cobalt electrolyte. In this work, two novel series of dyes were designed through modifying the π-conjugated bridge of the dyes D35 and DB (Chemistry structures, see Fig. 1). The furan (F), benzene ring (B) and benzo[*c*]thiophene (BT) units were introduced into the π-conjugated bridge of D35 in proper order, and the designed dyes were named as D35-1, D35-2 and D35-3, respectively. Moreover, by adjusting the position of DPP unit and the incorporation of fluorine in the conjugated bridge of DB, three novel dyes DB-1, DB-2 and DB-3 were designed. The work of Jia *et al*. had proved that the introduction of fluorine atom into the conjugated bridge of dye molecule can obviously improve the photoelectrical properties of DSSC^35^. With DFT and TD-DFT, the ground- and excited-state characteristics of the dyes, such as FMOs, energy gaps, ionization potentials (IPs), electron affinities (EAs), reorganization energies, spectra, charge transfer and the key parameters related to V_OC_ and J_SC_ were studied. Moreover, the effects of external electric field and dye aggregation on the photoelectrical and intermolecular charge transfer properties of the dyes were considered. The calculated results show that the designed dyes D35-3 and DB-2 could be used as a candidate for high performance dyes in the DSSC field due to their prominent photoelectrical and charge transfer characteristics.

## Results

### FMOs and energy gaps

**Figure 2:**
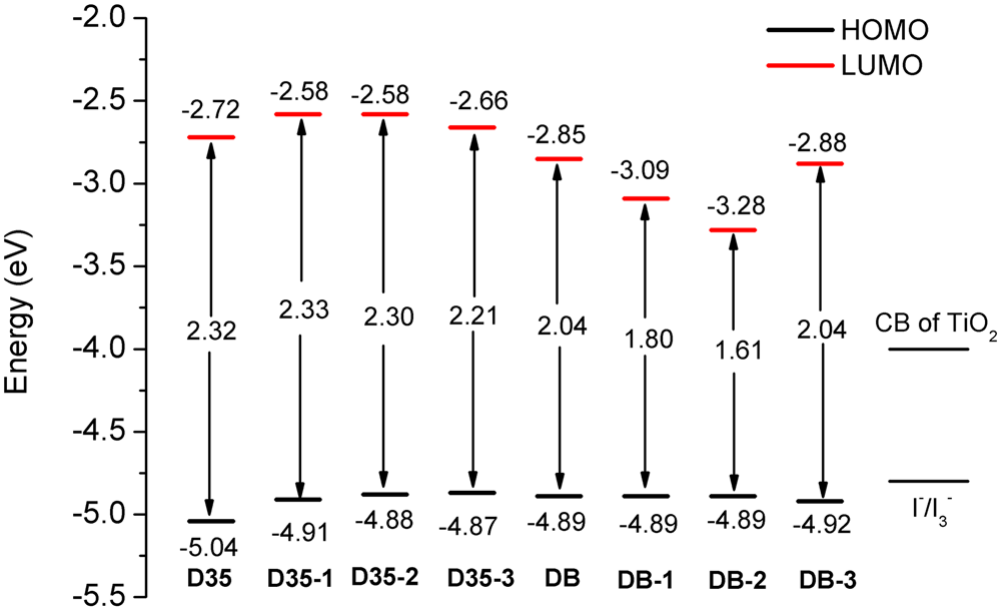
HOMO, LUMO energies and energy gaps of the original and designed dyes in acetonitrile.

The calculated energy levels and gaps of the original and designed dyes in acetonitrile are presented in Fig. 2. As shown, the HOMO energy level of DB (−4.89 eV) is higher than that of D35 (−5.04 eV), and the LUMO energy level (−2.85 eV) is below that of D35 (−2.72 eV), resulting in the lower energy gap of DB compared with that of D35. The results indicate that the introduction of DPP unit into the dye D35 has changed the energy levels and thereby improved the photoelectrical properties of DB. Noted that the HOMO energy levels for the original and designed molecules are not significantly different, and the largest and smallest HOMO energies are −4.87 eV and −5.04 eV for D35-3 and D35, respectively, implying that the modification of conjugated bridge for the dyes D35 and DB little affects the HOMO levels. However, it exhibits the obvious difference in the LUMO, i.e., DB-2 (−3.28 eV) < DB-1 (−3.09 eV) < DB-3 (−2.88 eV) < DB (−2.85 eV) < D35 (−2.72 eV) < D35-3 (−2.66 eV) < D35-1 = D35-2 (−2.58 eV). The results indicate that the energy levels of LUMO are more susceptible to be influenced by modification of conjugated bridge for the dyes D35 and DB.

In order to make the excited electron effectively injected into TiO_2_, dye higher LUMO is needed (here for TiO_2_ conduction band, usually −4.00 eV); for regeneration process, it is effectively restored for the oxidized dye under the lower HOMO compared with electrolyte (usually −4.80 eV for I^−^/I_3_^−^)^36,37^. As shown in Fig. 2, the higher LUMO energies for dyes and lower HOMO were found in comparison with the TiO_2_ and electrolyte, meaning the smooth completion of two processes (electron injection and dye regeneration).

Energy gap, defined as the difference between HOMO and LUMO levels, is a key factor affecting the solar cell PCE. A low energy gap is contributed to the better intramolecular charge transfer (ICT) and strong absorption band in spectra^38^. As shown in Fig. 2, it can be found that for D35 and its derivatives, the energy gaps are in the order of D35-3 (2.21 eV) < D35-2 (2.30 eV) < D35 (2.32 eV) < D35-1 (2.33 eV), indicating that the dye D35-3 would have a strong absorption band, and the introduction of benzo[*c*]thiophene unit into D35 should obviously improve the photoelectrical properties of dye. For DB and its derivatives, the energy gaps are DB-2 (1.61 eV) < DB-1 (1.80 eV) < DB = DB-3 (2.04 eV), and DB-2 exhibits the lowest energy gap among all the original and designed dyes, implying that DB-2 would show the best optical properties among the investigated dyes. The above results suggest that the photoelectrical properties of dye can be improved effectively by properly adjusting the position of the DPP unit in the conjugated bridge of DB, and the fluorine atoms has no obvious influence on the energy gap of DB molecule.

### Calculation on IPs and EAs

**Figure 3:**
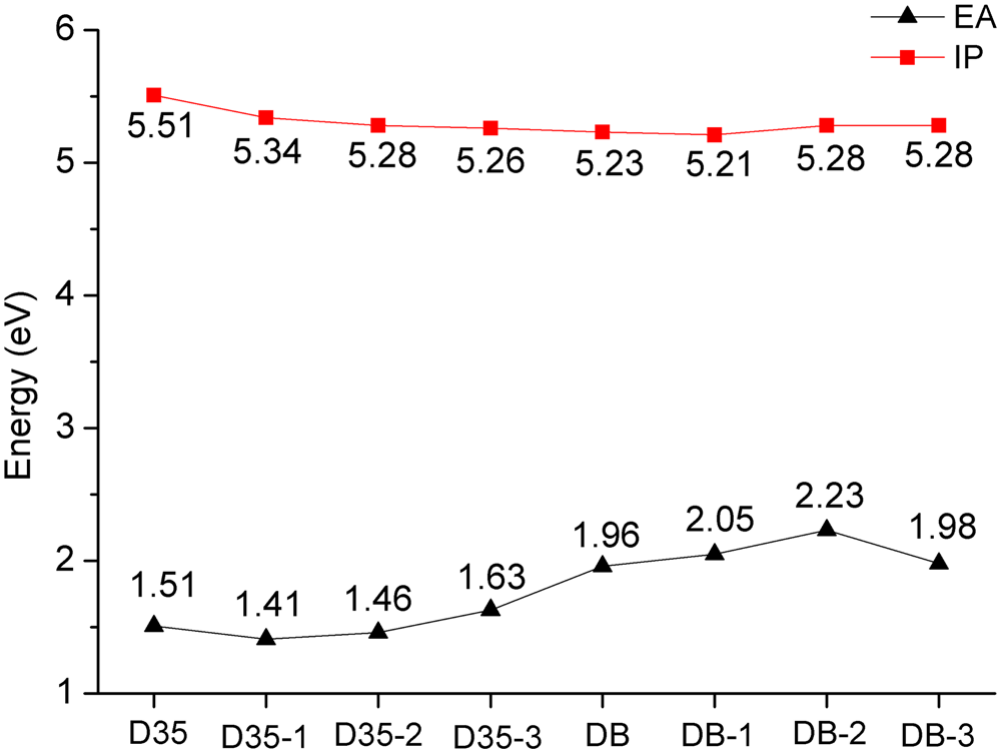
Ionization potentials (IPs) and electron affinities (EAs) of the original and designed dyes in acetonitrile.

Calculated IPs and EAs of the original and designed dyes are shown in Fig. 3. As shown in Fig. 3, the IP of DB (5.23 eV) is less than that of D35 (5.51 eV), indicating that the introduction of DPP unit into the dye D35 could result in the dye being easier to lose electrons, thereby making the dye DB exhibit better photoelectrical properties. Meanwhile, it can be found from Fig. 3 that the EA of DB (1.96 eV) is greater than that of D35, which means that introducing the DPP unit into the dye D35 could improve the electron accepting ability.

By comparing the IPs and EAs of the original and designed dyes, it can be found that there is no significant difference between the IPs of the original and designed dyes, and the largest and smallest IPs are 5.51 eV and 5.21 eV for D35 and DB-1, respectively. However, the calculated EAs of the original and designed dyes show a great difference, and the EAs of the original and designed dyes are DB-2 (2.23 eV) > DB-1 (2.05 eV) > DB-3 (1.98 eV) > DB (1.96 eV) > D35-3 (1.63 eV) > D35 (1.51 eV) > D35-2 (1.46 eV) > D35-1 (1.41 eV). The results imply that introducing the benzo[*c*]thiophene unit into the conjugated bridge of D35 and adjusting the position of DPP unit in the conjugated bridge of DB would improve the electron accepting ability of dyes, and the dye DB-2 would possess the best electron accepting ability among the investigated dyes.

### Excited state properties

**Figure 4:**
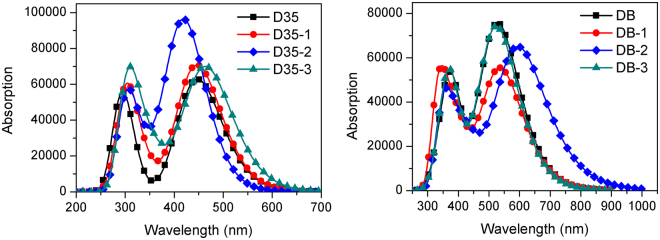
Simulated absorption spectra of the original and designed dyes in acetonitrile, in which: (**a**) for D35-series and (**b**) for DB-series.

**Figure 5:**
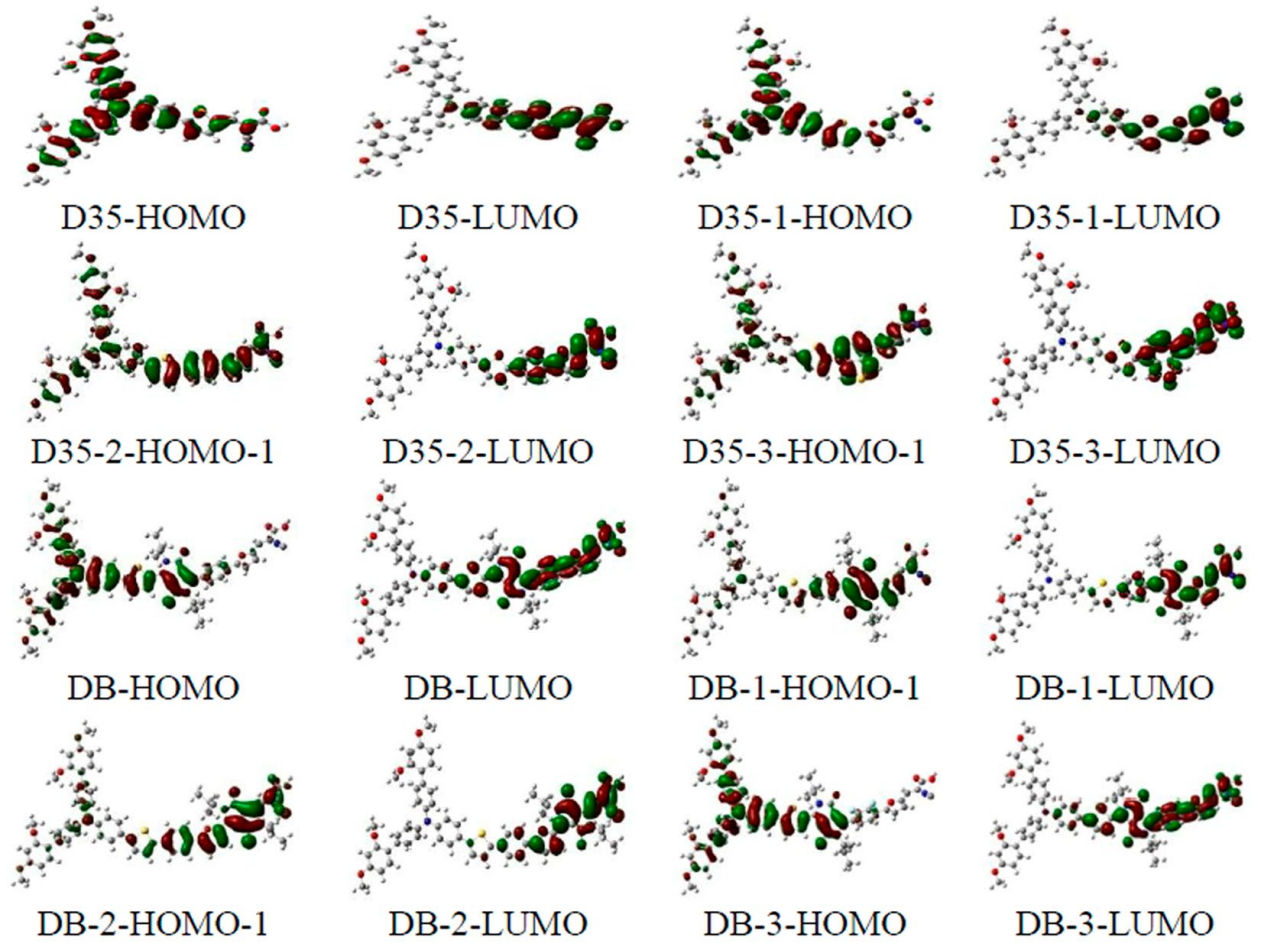
Diagrams of selected frontier molecular orbitals for the original and designed dyes.

Absorption spectra of the original and designed dyes in acetonitrile are presented in Fig. 4a and b. Table 1 shows the calculated peak site and oscillator strength (OS) as well as electron transition information. The absorption spectra of D35 and its derivatives with double-peaks characteristic are mainly distributed in the region of 250–550 nm (see Fig. 4a). The maximal absorption peaks corresponding to D35, D35-1, D35-2 and D35-3 are 447.49 nm, 446.63 nm, 420.34 nm and 464.27 nm, respectively, in which the maximal absorption peak corresponding to D35-1 appears a little change compared with that of D35. It is worth noting that the maximal absorption peaks corresponding to D35-3 and D35-2 exhibit a red and blue shift of 16.78 nm and 27.15 nm than D35, which implies that introduction of benzo[*c*]thiopheneorbenzene ring group into the conjugated bridge of D35-1 could result in the red or blue shift of absorption spectrum. As shown in Fig. 4a, it is interesting that the maximal peak OS of D35-2 is greater than that of D35, which is advantageous to the absorption of light. In addition, all the maximal peaks of D35 and its derivatives correspond to the S1 excited states, in which the S1 excited states of D35 and D35-1 correspond to the HOMO → LUMO transition. Those of D35-2 and D35-3 correspond to the HOMO-1 → LUMO. From the electron density in Fig. 5, the electron density of the above mentioned HOMO and HOMO-1 is distributed in whole molecules, and those of the LUMO levels reside in bridge and acceptor units of dyes, signifying ICT process for the dye D35 and its derivatives upon the photo-excitation.

**Table 1:** Calculated transition energies (*E*), absorption peaks (λ_abs_), dominant configuration coefficients and oscillator strengths (*f*) of the original and designed dyes in acetonitrile.

Dye	State	*E* (eV)	${{\rm{\lambda }}}_{{\rm{abs}}}$ (nm)	Contribution MO	Strength *f*
D35	S1	2.7706	447.49	(0.61188)H → L	1.5501
	S2	3.8347	323.32	(0.48128)H-2 → L	0.0406
	S3	4.1499	298.76	(0.64108)H → L + 2	1.0020
	S4	4.3212	286.92	(0.47702)H → L + 1	0.2177
	S5	4.3864	282.66	(0.48637)H → L + 3	0.1515
	S6	4.5231	274.11	(0.42922)H-10 → L	0.1007
D35-1	S1	2.7760	446.63	(0.54737)H → L	1.7466
	S2	3.6520	339.50	(0.46689)H-1 → L	0.2968
	S3	4.0653	304.98	(0.48377)H → L + 1	0.3029
	S4	4.0927	302.94	(0.61580)H → L + 2	1.0280
	S5	4.3255	286.63	(0.53834)H → L + 4	0.0677
	S6	4.5148	274.62	(0.43880)H-1 → L + 1	0.0809
D35-2	S1	2.9496	420.34	(0.46287)H-1 → L	2.3351
	S2	3.5426	349.98	(0.44392)H → L + 1	0.3912
	S3	4.0395	306.93	(0.50921)H → L	0.2765
	S4	4.0757	304.21	(0.62009)H → L + 2	1.0291
	S5	4.2405	292.38	(0.43802)H-1 → L + 1	0.0186
	S6	4.3216	286.90	(0.62299)H → L + 4	0.0376
D35-3	S1	2.6705	464.27	(0.47786)H-1 → L	1.7158
	S2	3.4393	360.49	(0.35964)H-1 → L	0.4557
	S3	3.6914	335.87	(0.47365)H-1 → L + 1	0.1143
	S4	3.9375	314.88	(0.42545)H → L + 2	0.5760
	S5	4.0795	303.92	(0.59347)H → L + 3	0.9879
	S6	4.1200	300.93	(0.46043)H-4 → L	0.0922
DB	S1	2.3443	528.88	(0.55542)H → L	1.8625
	S2	3.1325	395.80	(0.44525)H-1 → L	0.0748
	S3	3.2921	376.62	(0.36687)H-1 → L + 1	1.1391
	S4	3.5606	348.21	(0.61062)H-8 → L	0.0056
	S5	3.6857	336.40	(0.37450)H → L + 1	0.2725
	S6	4.0371	307.11	(0.28132)H → L + 2	0.0652
DB-1	S1	2.3070	537.43	(0.60799)H-1 → L	1.3544
	S2	3.0942	400.70	(0.43265)H → L	0.6467
	S3	3.4729	357.00	(0.57717)H-9 → L	0.0656
	S4	3.4960	354.65	(0.46730)H-1 → L + 1	0.1810
	S5	3.6655	338.24	(0.39180)H → L + 2	1.0237
	S6	3.8252	324.12	(0.43949)H-5 → L	0.0497
DB-2	S1	2.0807	595.89	(0.59792)H-1 → L	1.5957
	S2	2.8850	429.76	(0.44994)H → L	0.5840
	S3	3.3317	372.13	(0.67007)H-10 → L	0.0242
	S4	3.3771	367.14	(0.40551)H → L	0.3754
	S5	3.5051	353.72	(0.38934)H → L + 1	0.6189
	S6	3.7059	334.56	(0.47496)H-1 → L + 1	0.1060
DB-3	S1	2.3635	524.58	(0.53881)H → L	1.8272
	S2	3.1381	395.10	(0.47089)H-1 → L	0.0478
	S3	3.3154	373.97	(0.36254)H-1 → L + 1	1.1684
	S4	3.5436	349.88	(0.60823)H-8 → L	0.0039
	S5	3.6714	337.70	(0.36896)H → L + 1	0.2927
	S6	4.0577	305.55	(0.48684)H-15 → L	0.0286

Simultaneously, it can be found from Fig. 4b that the absorption spectra of DB and its derivatives show a good response to the solar spectrum, which covers almost the whole visible and even infrared light region. The absorption spectra of DB and its derivatives also show the double-peak characteristic, which is similar to the shapes of absorption spectra of D35 and its derivatives. Table 1 shows that absorption peaks are DB-2 (595.89 nm) > DB-1 (537.43 nm) > DB (528.88 nm) > DB-3 (524.58 nm), in which the maximal absorption peak of DB-2 shows the greatest bathochromic-shift of 67.01 nm compared with that of DB. In addition, it can be found that all the maximal absorption peaks of DB and its derivatives also correspond to the S1 excited states (HOMO → LUMO transition), and that of DB-1 and DB-2 is HOMO-1 → LUMO. The electron density indicates that upon the photo-excitation, the significant ICT occurs in the DB and DB-3 molecules (see Fig. 5).

**Figure 6:**
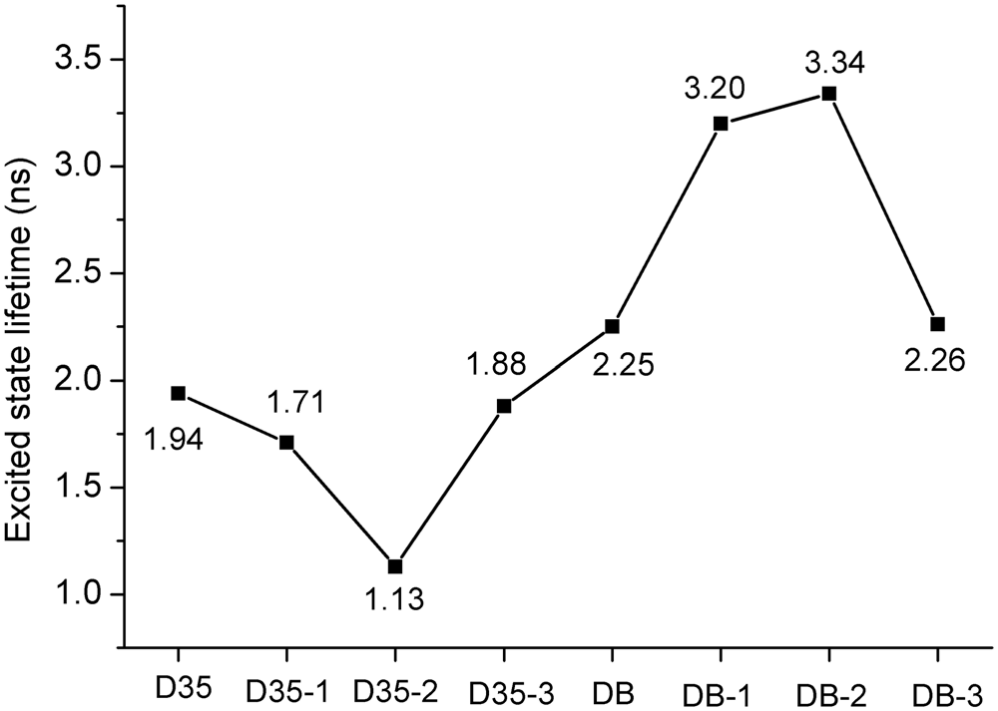
Calculated first excited state lifetimes for the original and designed dyes.

The dye with longer excited state lifetime would behave with higher charge transfer efficiency^38^, and the excited state lifetime of dye is estimated by using the following equation^39^:

1 $${\rm{\tau }}=\frac{1.499}{f\times {E}^{2}}$$

where *E* stands for the excitation energy corresponding to different excited state, and *f* represents the excited state OS. The calculated first excited lifetimes of the original and designed dyes are presented in Fig. 6. It was found intuitively from Fig. 6 that the dye DB-2 exhibits the largest first excited-state lifetime among all the investigated dyes, and the calculated first excited-state lifetimes are in this order: DB-2 > DB-1 > DB-3 > DB > D35 > D35-3 > D35-1 > D35-2. Moreover, the dye D35-3 shows the longest excited state lifetime among the designed dyes based on D35. In summary, DB-2 and D35-3 could be used as the candidates for high efficiency dye due to their excellent optical properties among the investigated dye molecules.

ICT is accompanied by the photo-excitation, and the excited state with highly efficient charge separation properties would be propitious to the ultrafast interfacial electron injection and reducing the electrons recombination rate^40^. Table 2 shows the parameters contain the charge-transfer length (${{\rm{D}}}_{{\rm{CT}}}$), transferred charge (${\rm{\Delta }}q$), the half of the sum of two centroid axis along the electron transfer direction (*H*), the difference between *H* and ${D}_{{\rm{CT}}}$ (*t*) and the exciton binding energy (${E}_{{\rm{b}}}$), in which the greater *t* results in the better separation between the density increment and depletion regions^41^.

**Table 2:** Calculated *D*_CT_, ∆q, *H*, *t* and *E*_b_ for the original and designed dyes.

Dye	${{\boldsymbol{D}}}_{{\bf{CT}}}$ (Å)	∆q (e)	*H* (Å)	*t* (Å)	${E}_{{\rm{b}}}$(eV)
D35	2.73	0.77	6.30	3.57	0.45
D35-1	2.51	0.70	7.40	4.89	0.45
D35-2	2.72	0.71	8.91	6.19	0.65
D35-3	2.19	0.72	8.70	6.51	0.46
DB	1.03	0.74	9.34	8.31	0.30
DB-1	1.22	0.75	10.30	9.08	0.51
DB-2	1.06	0.78	10.61	9.55	0.47
DB-3	1.23	0.76	9.33	8.10	0.32

**Figure 7:**
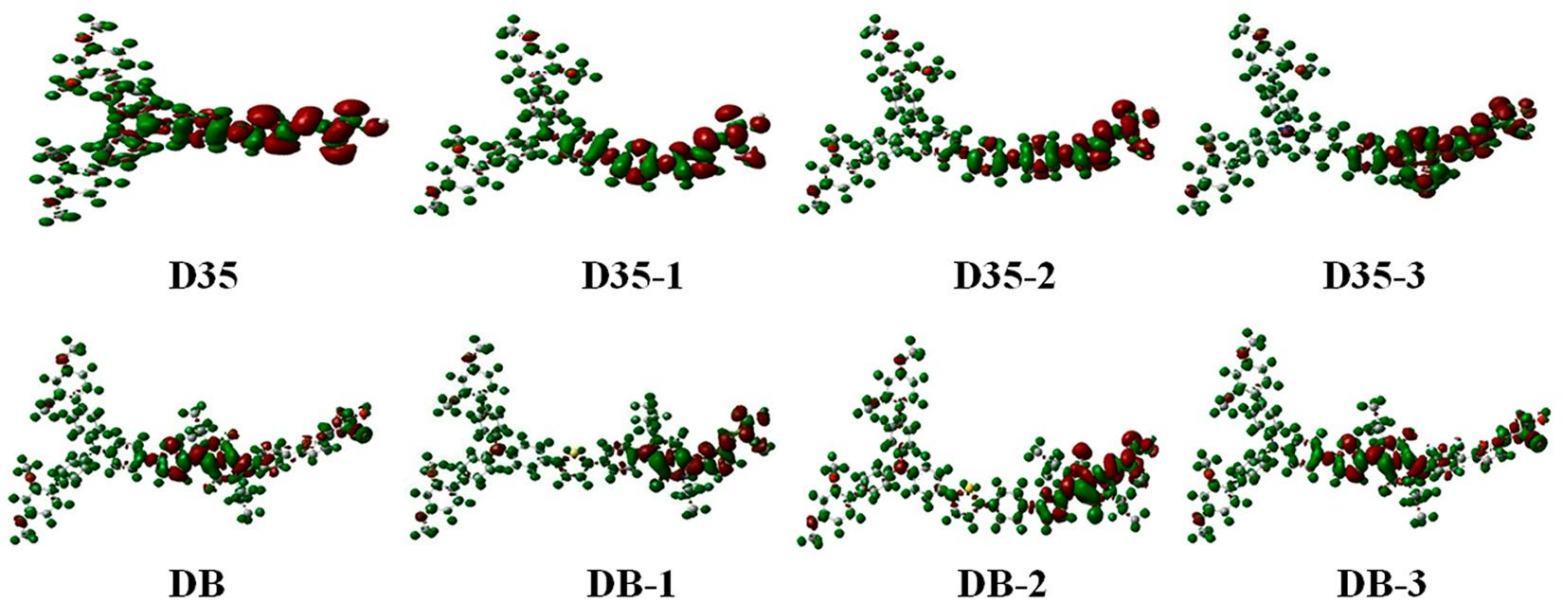
Calculated charge density difference between the ground and excited states of the original and designed dyes, in which the green and red regions represent the lessen and increase areas of electron, respectively.

The average ${\rm{\Delta }}q$ for dye DB and its derivatives (0.76 e) is greater than those for the dye D35 and its derivatives (0.73 e), indicating that the ICT is more likely to occur in the dye system based on DB compared with the dye system based on D35. For the dye D35 and its derivatives, the ${\rm{\Delta }}q$ are in the order of D35 > D35-3 > D35-2 > D35-1, implying that the dye D35-3 would exhibit the better ICT properties compared with the other derivatives of D35. For the dye DB and its derivatives, the obtained ${\rm{\Delta }}q$ follows the order of DB-2 > DB-3 > DB-1 > DB, which suggests that the dye DB-2 would have the best ICT characteristics among the dye DB and its derivatives. The obtained charge density difference is presented in Fig. 7, from which can be seen clearly that there is ICT process. In addition, from Table 2 it can be found that the obtained *t* for all the original and designed dyes are in the order of DB-2 > DB-1 > DB > DB-3 > D35-3 > D35-2 > D35-1 > D35, meaning that the dye DB and its derivatives would show the better charge separation compared with the dye D35 and its derivatives. Moreover, the dyes D35-3 and DB-2 show the largest *t* value among the dyes D35 and DB series, respectively, which signifies that the dyes D35-3 and DB-2 would present the best charge separation among the two dye series.

The exciton should be generated immediately as long as the ICT occurs under the photo-excitation. In order to effectively separate the exciton, the energy of exciton binding (${E}_{{\rm{b}}}$) must be overcome, which can be estimated by electronic and optical band gap^42^. Table 2 shows that the calculated ${E}_{{\rm{b}}}$ for the original and designed dyes, i.e., the average ${E}_{{\rm{b}}}$ value for the dye DB and its derivatives (0.40 eV) is lower than that for the dye D35 and its derivatives (0.50 eV), indicating that the excitons in the dye DB and its derivatives are easier to separate compared with that in the dye D35 and its derivatives.

### Emission characteristics

Table 3 shows the obtained emission peaks, OS and radiative lifetimes of the original and designed dyes. All the emission peaks corresponding to S1 state are composed of HOMO → LUMO transition except for the dyes DB-1 and DB-2. For the dye D35 and its derivatives, the emission peaks are D35-3 (565.09 nm) > D35-1 (504.91 nm) > D35 (486.32) ≈ D35-2 (486.24 nm), and the Stokes shifts are 38.83 nm, 58.28 nm, 65.90 nm and 100.82 nm for D35, D35-1, D35-2 and D35-3, respectively, in which D35-3 exhibits the largest emission peak and Stokes shift among the four dyes. For the dye DB and its derivatives, the emission peaks are in the order of DB-2 (650.26 nm) > DB (627.66 nm) > DB-1 (607.69 nm) ≈ DB-3 (605.80 nm), and the Stokes shifts for DB, DB-1, DB-2 and DB-3 are 98.78 nm, 70.26 nm, 54.37 nm and 81.22 nm, respectively, in which the dyes DB-2 and DB show the largest emission peak and Stokes shift among the four dyes, respectively. The large Stokes shift maybe due to the significant geometry deform on going from the ground state to excited state of the dye molecule^43^. Among the original and designed dyes, DB-2 and D35-3 show the largest emission peak and Stokes shift, respectively.

**Table 3:** Calculated emission energies (*E*), emission peaks (λ_em_), oscillator strengths (*f*) and radiative lifetimes (*τ*) of the original and designed dyes in acetonitrile.

Dye	State	*E* (eV)	λ_em_ (nm)	Contribution MO	Strength *f*	*τ* (ns)
D35	S1	2.5494	486.32	(0.61368)H → L	1.0063	3.52
D35-1	S1	2.4556	504.91	(0.56236)H → L	1.7861	2.14
D35-2	S1	2.5498	486.24	(0.50189)H → L	2.4133	1.47
D35-3	S1	2.1941	565.09	(0.57367)H → L	1.8781	2.55
DB	S1	1.9753	627.66	(0.62412)H → L	2.1175	2.79
DB-1	S1	2.0403	607.69	(0.58659)H-1 → L	1.5725	3.52
DB-2	S1	1.9067	650.26	(0.62852)H-1 → L	1.6616	3.82
DB-3	S1	2.0466	605.80	(0.59131)H → L	2.1025	2.62

In addition, the radiative lifetimes of the original and designed dyes were estimated from the following equation^44^:

2 $${\rm{\tau }}=\frac{{{\rm{c}}}^{3}}{2{({{\rm{E}}}_{{\rm{Flu}}})}^{2}{\rm{f}}}$$

where c represents the velocity of light; *E*_Flu_ is the emission energy and *f* stands for the OS. Table 3 displays that the radiative lifetimes are in the order of D35 (3.52 ns) > D35-3 (2.55 ns) > D35-1 (2.14 ns) > D35-2 (1.47 ns), in which D35-3 exhibits the longest radiative lifetime among the derivatives of D35; for the dye DB and its derivatives, the radiative lifetimes are DB-2 (3.82 ns) > DB-1 (3.52 ns) > DB (2.79 ns) > DB-3 (2.62 ns). The above results indicate that the dye DB-2 possesses the longest radiative lifetime among the original and designed dyes.

### Reorganization energies

**Figure 8:**
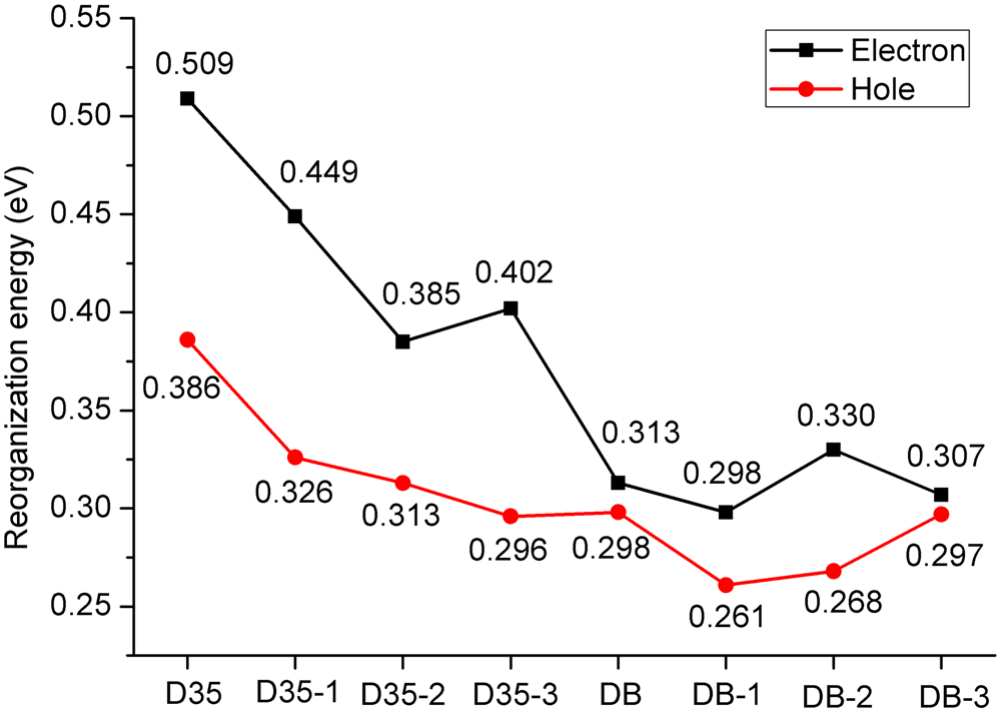
Calculated electron and hole reorganization energies of the original and designed dyes.

The dye molecule with prominent performance should possess the good charge transfer rate, and the charge transfer rate arose from the standard Marcus/Hush model expressed as follows^45^:

3 $${\rm{\kappa }}={(\frac{{\rm{\pi }}}{\lambda {{\rm K}}_{{\rm{b}}}T})}^{1/2}\frac{{V}^{2}}{\hslash }\exp (-\frac{\lambda }{4{{\rm K}}_{{\rm{b}}}T})$$

where ${{\rm K}}_{{\rm{b}}}$ stands for the Boltzmann constant, *T* represents the temperature, *V* is the electronic coupling matrix element between the two species, and ${\rm{\lambda }}$ is the reorganization energy, which contains the inter- and intra-molecular reorganization energy^46^. However, the intermolecular reorganization energy has no significant effect on the electron transfer, so the intramolecular reorganization energy was only focused on in this work, which can be calculated as follows^47^:

4 $${{\rm{\lambda }}}_{{\rm{e}}}=({{\rm{E}}}_{0}^{-}-{{\rm{E}}}_{-})+({{\rm{E}}}_{-}^{0}-{{\rm{E}}}_{0})$$

5 $${{\rm{\lambda }}}_{{\rm{h}}}=({{\rm{E}}}_{0}^{+}-{{\rm{E}}}_{+})+({{\rm{E}}}_{+}^{0}-{{\rm{E}}}_{0})$$

where ${{\rm{E}}}_{0}$, ${{\rm{E}}}_{0}^{+}$(${{\rm{E}}}_{0}^{-}$) ${\mathrm{and}{\rm{E}}}_{+}$(${{\rm{E}}}_{-}$) represent neutral molecule optimized energy, charged molecular energy on the basis of neutral and charged ground state, respectively. Figure 8 displays that all the hole reorganization energies are lower than the electron reorganization energies of the investigated dyes, indicating that the investigated dyes have a better hole transport ability. For the dye D35 and its derivatives, the electron and hole reorganization energies are D35-2 < D35-3 < D35-1 < D35 and D35-3 < D35-2 < D35-1 < D35, respectively, implying that the dyes D35-2 and D35-3 have the better electron and hole transfer ability among the dye D35 and its derivatives. For the dye DB and its derivatives, the electron and hole reorganization energies follow the order of DB-1 < DB-3 < DB < DB-2 and DB-1 < DB-2 < DB-3 < DB, respectively, which indicates that the dye DB-1 would exhibit the best electron and hole transfer ability among the dye DB and its derivatives. Moreover, as shown in Fig. 8, DB-1 has the lowest reorganization energies among original and designed dyes, meaning that DB-1 would show the best charge transfer ability among the investigated dyes. In addition, it is worth noting from Fig. 8 that the electron and hole reorganization energies of DB and DB-3 show the lowest difference compared with that of the other dyes, indicating that the two dyes have the better charge transfer balance performance among the investigated dyes.

### Key parameters associated with V_oc_ and J_sc_

The PCE (${\rm{\eta }}$) of DSSC is determined by the open circuit voltage (${{\rm{V}}}_{{\rm{OC}}}$), the short-circuit current density (${{\rm{J}}}_{{\rm{SC}}}$) and the fill factor (FF), which can be expressed as following^48^:

6 $${\rm{\eta }}={\rm{FF}}\frac{{{\rm{V}}}_{{\rm{OC}}}{{\rm{J}}}_{{\rm{SC}}}}{{{\rm{P}}}_{{\rm{inc}}}}$$

where ${{\rm{P}}}_{{\rm{inc}}}$ represents the incident light intensity. The V_oc_ is defined as the difference between the Fermi level of the semiconductor (usually TiO_2_) and the redox potential of the electrolyte (usually ${{\rm{I}}}^{-}/{{\rm{I}}}_{3}^{-}$)^49^:

7 $${{\rm{V}}}_{{\rm{OC}}}={{\rm{E}}}_{{{\rm{TiO}}}_{2}}-{{\rm{E}}}_{{{\rm{I}}}^{-}/{{\rm{I}}}_{3}^{-}}=\frac{{{\rm{E}}}_{{\rm{CB}}}+{{\rm{\Delta }}E}_{{\rm{CB}}}}{{\rm{q}}}+\frac{{{\rm{\kappa }}}_{{\rm{B}}}{\rm{T}}}{{\rm{q}}}\,\mathrm{ln}\,\frac{{{\rm{n}}}_{{\rm{c}}}}{{{\rm{N}}}_{{\rm{CB}}}}-\frac{{{\rm{E}}}_{{\rm{redox}}}}{{\rm{q}}}$$

where ${{\rm{E}}}_{{\rm{CB}}}$ is the conduction band (CB) of the semiconductor, ${{\rm{\Delta }}{\rm{E}}}_{{\rm{CB}}}$ represents the shift of the CB of semiconductor, q is the elementary charge, ${{\rm{\kappa }}}_{{\rm{B}}}$ is the Boltzmann constant, $T$ is the temperature, ${{\rm{N}}}_{{\rm{CB}}}$ is the effective density of state, ${{\rm{n}}}_{{\rm{c}}}$ is the number of electrons in the CB of semiconductor, and ${{\rm{E}}}_{{\rm{redox}}}$ is the redox potential of the electrolyte (usually −4.80 eV for ${{\rm{I}}}^{-}/{{\rm{I}}}_{3}^{-}$)^50^. ${{\rm{\Delta }}{\rm{E}}}_{{\rm{CB}}}$ can be expressed as following^51^:

8 $${{\rm{\Delta }}{\rm{E}}}_{{\rm{CB}}}=\frac{-{q{\rm{\mu }}}_{{\rm{normal}}}{\rm{\gamma }}}{{{\rm{\varepsilon }}}_{0}{\rm{\varepsilon }}}$$

where ${{\rm{\mu }}}_{{\rm{normal}}}$, ${\rm{\gamma }}$, ${{\rm{\varepsilon }}}_{0}$(ε) are the dipole moment, the coverage of dye absorbed on the TiO_2_ and the dielectric constant, respectively. As can be seen from the equations (7) and (8) that the greater ${{\rm{\mu }}}_{{\rm{normal}}}$ and ${{\rm{\Delta }}{\rm{E}}}_{{\rm{CB}}}$ of dye would emerge, ${{\rm{V}}}_{{\rm{OC}}}$ is the larger. The density of state (DOS) and partial DOS (PDOS) charts of the dye/TiO_2_ complexes, related to the ${{\rm{\Delta }}E}_{{\rm{CB}}}$, are shown in Fig. S1 (see Supplementary Fig. S1), and Table 4 shows the calculated ${{\rm{\mu }}}_{{\rm{normal}}}$ and ${{\rm{\Delta }}{\rm{E}}}_{{\rm{CB}}}$ of the original and designed dyes. For original and designed dyes, the ${{\rm{\mu }}}_{{\rm{normal}}}$ and ${{\rm{\Delta }}{\rm{E}}}_{{\rm{CB}}}$ are in the order of DB-2 > D35-1 > D35-2 > D35-3 > D35 > DB-3 > DB > DB-1 and DB > DB-3 > DB-1 = DB-2 > D35-3 > D35-2 > D35-1 > D35. The above results indicate that the strategy of modification of different groups in the π-conjugated bridge or adjusting the position of the DPP unit in the π-conjugate bridge could improve the ${{\rm{V}}}_{{\rm{OC}}}$ of the dyes.

**Table 4:** Calculated driving force of electron injection (∆G^inject^), dipole moment (μ_normal_), light harvesting efficiency (LHE) and shift of E_CB_ (∆E_CB_) of the original and designed dyes.

Dye	${{\bf{E}}}_{{\bf{OX}}}^{{\bf{dye}}}$ (eV)	${{\bf{E}}}_{{\bf{OX}}}^{{\bf{dye}}{\boldsymbol{\ast }}}$ (eV)	∆G^inject^ (eV)	LHE	μ_normal_ (D)	∆E_CB_ (eV)
D35	−5.04	−2.27	−1.73	0.9718	7.8435	0.37
D35-1	−4.91	−2.13	−1.87	0.9821	9.6140	0.39
D35-2	−4.88	−1.93	−2.07	0.9954	8.5047	0.40
D35-3	−4.87	2.20	−1.80	0.9808	8.3303	0.47
DB	−4.89	2.55	−1.45	0.9863	7.1723	0.74
DB-1	−4.89	2.58	−1.42	0.9558	4.7844	0.57
DB-2	−4.89	2.81	−1.19	0.9746	10.6938	0.57
DB-3	−4.92	2.56	−1.44	0.9851	7.6900	0.68

In addition, ${{\rm{J}}}_{{\rm{SC}}}$ is mainly depended on the LHE, the inject efficiency ${{\rm{\Phi }}}_{{\rm{inj}}}$ and collection efficiency ${{\rm{\eta }}}_{{\rm{coll}}}$ according to the following relationship^41,52^:

9 $${{\rm{J}}}_{{\rm{SC}}}={\rm{e}}{\int }^{}{\rm{LHE}}({\rm{\lambda }}){{\rm{\Phi }}}_{{\rm{inj}}}{{\rm{\eta }}}_{{\rm{coll}}}{{\rm{I}}}_{0}({\rm{\lambda }})d{\rm{\lambda }}$$

For the same DSSC, ${{\rm{\eta }}}_{{\rm{coll}}}$ can be considered as a constant. Therefore, the ${{\rm{J}}}_{{\rm{SC}}}$ is determined only by the factors of ${\rm{LHE}}$ and ${{\rm{\Phi }}}_{{\rm{inj}}}$, which are light absorption efficiency and injection efficiency of electron. The LHE is correlated with the calculated oscillator strength (*f*), which is represented as^53^: ${\rm{LHE}}=1-{10}^{-{\rm{f}}}$. The ${{\rm{\Phi }}}_{{\rm{inj}}}$ is positively correlated with the driving force of electron injection (${{\rm{\Delta }}{\rm{G}}}^{{\rm{inject}}}$), which can be defined as^54,55^: ${{\rm{\Delta }}{\rm{G}}}^{{\rm{inject}}}={{\rm{E}}}_{{\rm{OX}}}^{\mathrm{dye}\ast }-{{\rm{E}}}_{{\rm{CB}}}$; Table 4 shows the calculated ${\rm{LHE}}$, ${{\rm{E}}}_{{\rm{OX}}}^{\text{dye}\ast }$ and ${{\rm{\Delta }}{\rm{G}}}^{{\rm{inject}}}$. The obtained LHE is in the range of 0.9558–0.9954, which exhibits no obvious difference. Moreover, the calculated values of ${{\rm{\Delta }}{\rm{G}}}^{{\rm{inject}}}$ for all molecules are far greater than 0.2 eV, which means the sufficient driving force is provided to fulfill electron injection process^56^. In addition, the relatively lower ${E}_{{\rm{b}}}$ can generate the better ${{\rm{\Phi }}}_{{\rm{inj}}}$^41^. As listed in Table 2, the obtained ${E}_{{\rm{b}}}$ for the dye D35 and DB are 0.45 eV and 0.30 eV, respectively. The results indicate that the dye DB would have a better ${{\rm{\Phi }}}_{{\rm{inj}}}$, thereby showing the better photoelectrical performance, which is consistent with the experimental results^34^. The average value of ${E}_{{\rm{b}}}$ for the dye DB and its derivatives (0.40 eV) is lower than that for the dye D35 and its derivatives (0.50 eV), implying that the dye DB and its derivatives would show the better ${{\rm{\Phi }}}_{{\rm{inj}}}$.

### Excited state properties of dye/TiO_2_ complexes

**Figure 9:**
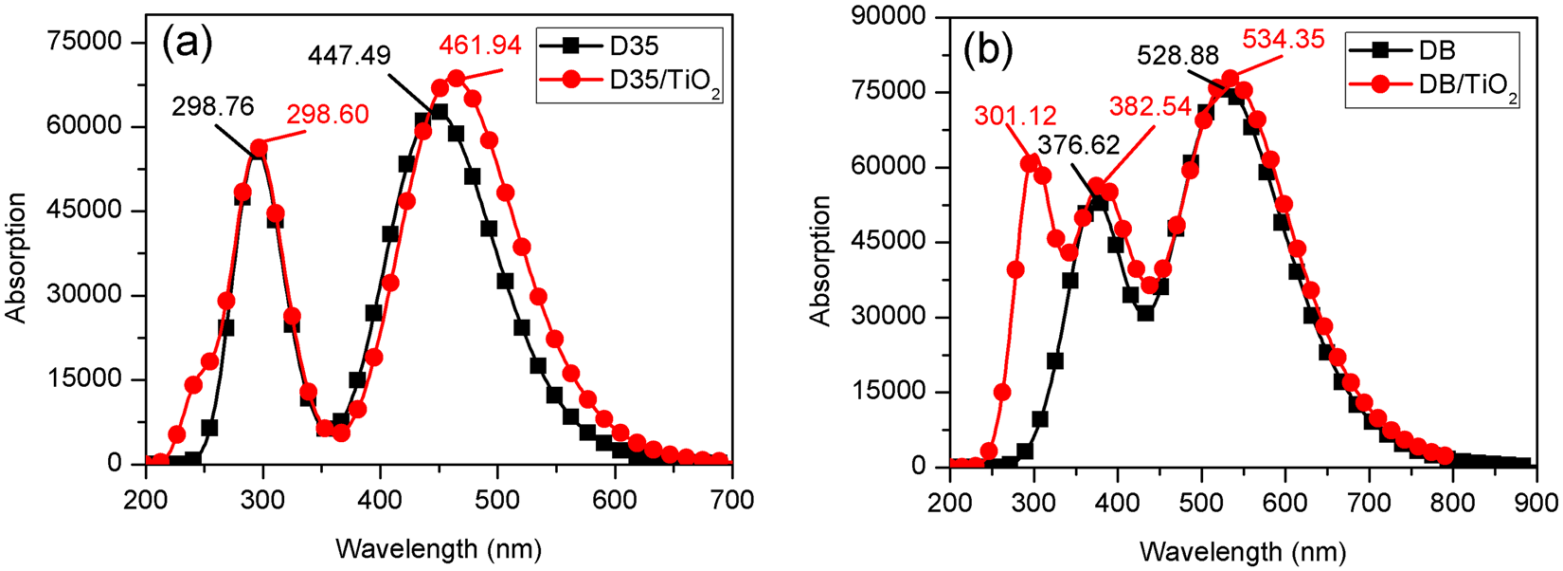
Simulated absorption spectra of (**a**) isolated D35 and D35/TiO_2_ complex; and (**b**) isolated DB and DB/TiO_2_ complex.

For understanding the dyes and TiO_2_ interaction, the structure and CT properties of the dye/TiO_2_ complexes were investigated. Table 5 and Table S1-S2 (see Supplementary Table S1-S2) show the excited information about ten excited states of the dye/TiO_2_ complexes. The simulated absorption spectra of the dye/TiO_2_ complexes are presented in Fig. 9 and Fig. S2 (see Supplementary Fig. S2). Figure 9 and Fig. S2 show the spectra of DB/TiO_2_ and DB-3/TiO_2_ complexes emerge a new absorption band (301.12 nm and 300.67 nm for DB/TiO_2_ and DB-3/TiO_2_, respectively). Except for the DB/TiO_2_ and DB-3/TiO_2_ complexes, the absorption spectra of the other complexes do not show extra absorption band in comparison with the isolated dyes.

**Table 5:** Calculated excited state properties of the dye/TiO_2_ complexes in acetonitrile.

Dye	State	*E* (eV)	λ_abs_ (nm)	Contribution MO	Strength *f*
D35	S1	2.6840	461.94	(0.60922)H → L	1.6968
	S3	4.1521	298.60	(0.63755)H → L + 5	1.0029
	S4	4.2747	290.04	(0.50927)H → L + 4	0.2127
D35-1	S1	2.7019	458.87	(0.53659)H → L	1.8019
	S3	3.9834	311.25	(0.42402)H → L + 4	0.3601
	S4	4.0856	303.47	(0.61906)H → L + 5	1.0099
D35-2	S1	2.8784	430.74	(0.46572)H-1 → L	2.3658
	S2	3.4906	355.19	(0.34811)H → L + 4	0.4663
	S4	4.0690	304.70	(0.62221)H → L + 5	1.0344
D35-3	S1	2.6193	473.34	(0.48220)H-1 → L	1.7930
	S4	3.8859	319.06	(0.40318)H → L + 5	0.6266
	S5	4.0713	304.53	(0.58023)H → L + 6	0.8836
DB	S1	2.3203	534.35	(0.53812)H → L	1.9103
	S3	3.2411	382.54	(0.37754)H-1 → L + 1	1.0609
	S8	4.1174	301.12	(0.53977)H → L + 6	0.9092
DB-1	S1	2.2824	543.22	(0.60089)H-1 → L	1.3952
	S5	3.6365	340.95	(0.35083)H → L + 5	1.1184
	S9	4.0773	304.09	(0.61121)H → L + 6	1.0624
DB-2	S1	2.1036	589.40	(0.60382)H-1 → L	1.6226
	S2	2.9256	423.79	(0.44444)H → L	0.6401
	S8	4.0742	304.32	(0.60024)H → L + 6	1.0770
DB-3	S1	2.3537	526.76	(0.52610)H → L	1.8537
	S3	3.2710	379.04	(0.37092)H-1 → L + 1	1.2683
	S9	4.1235	300.67	(0.56969)H → L + 6	0.9992

The selected transition properties corresponding to the first three absorption peaks of the dye/TiO_2_ complexes are listed in Table 5. As shown, the maximal absorption peaks of all the complexes correspond to the S1 excited state. For the dye D35 and its derivatives, the S1 excited states for D35/TiO_2_, D35-1/TiO_2_, D35-2/TiO_2_ and D35-3/TiO_2_ originate from H → L, H → L, H-1 → L and H-1 → L, respectively. The maximal absorption peaks for D35/TiO_2_, D35-1/TiO_2_, D35-2/TiO_2_ and D35-3/TiO_2_ are 461.94 nm, 458.87 nm, 430.74 nm and 473.34 nm, which show the red-shift of 14.45 nm, 12.24 nm, 10.40 and 9.07 nm compared with that for the corresponding isolated dyes, respectively. For the dye DB and its derivatives, the S1 excited states for DB/TiO_2_, DB-1/TiO_2_, DB-2/TiO_2_ and DB-3/TiO_2_are composed of H → L, H-1 → L, H-1 → L and H → L transitions. The maximal peaks are 534.35 nm, 543.22 nm, 589.40 nm and 526.76 nm for DB/TiO_2_, DB-1/TiO_2_, DB-2/TiO_2_ and DB-3/TiO_2_, which have the red-shift of 5.47 nm, 5.79 nm and 2.18 nm compared with that for the isolated dye DB, DB-1 and DB-3, respectively.

**Figure 10:**
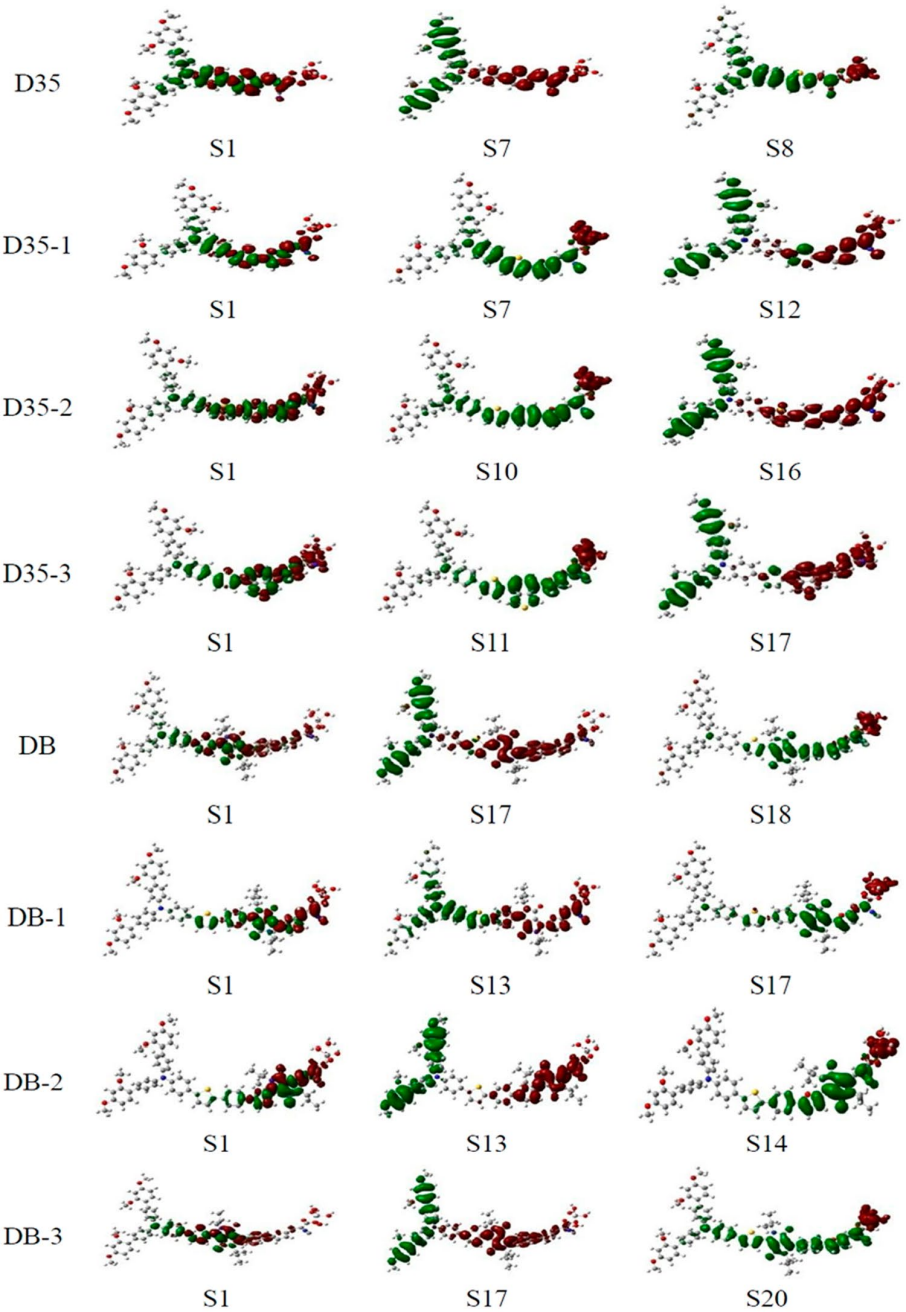
Selected charge density difference (CDD) diagrams of the dye/TiO_2_ complexes.

To investigate the electron transfer process in the excited states of dye/TiO_2_ complexes, the charge density difference (CDD) diagrams corresponding to some excited states of the dye/TiO_2_ complexes are shown in Fig. 10. As illustrated in Fig. 10, taking the D35/TiO_2_ complex as an example, the S1 excited state belongs to a local excitation state, for which the electrons and holes are distributed over the complex alternately. Similarly, it can be found from Fig. S3 (see Supplementary Fig. S3) that the excited states S2–S6, S10, S13–S16, S18–S20 are attributed to the local excited state. In addition, the holes and electrons for the S7 are entirely separated in complex system, which can be vested in the charge-transfer excitation and is similar to the charge distribution of the excited states S11 and S17 (See Supplementary Fig. S3). It is interesting that for the excited state S8, the holes and electrons are fully distributed in the dye and TiO_2_ cluster, respectively, which is similar to the charge distribution of the excited states S9 and S12 (See Supplementary Fig. S3).

### Chemical reactivity parameters

**Figure 11:**
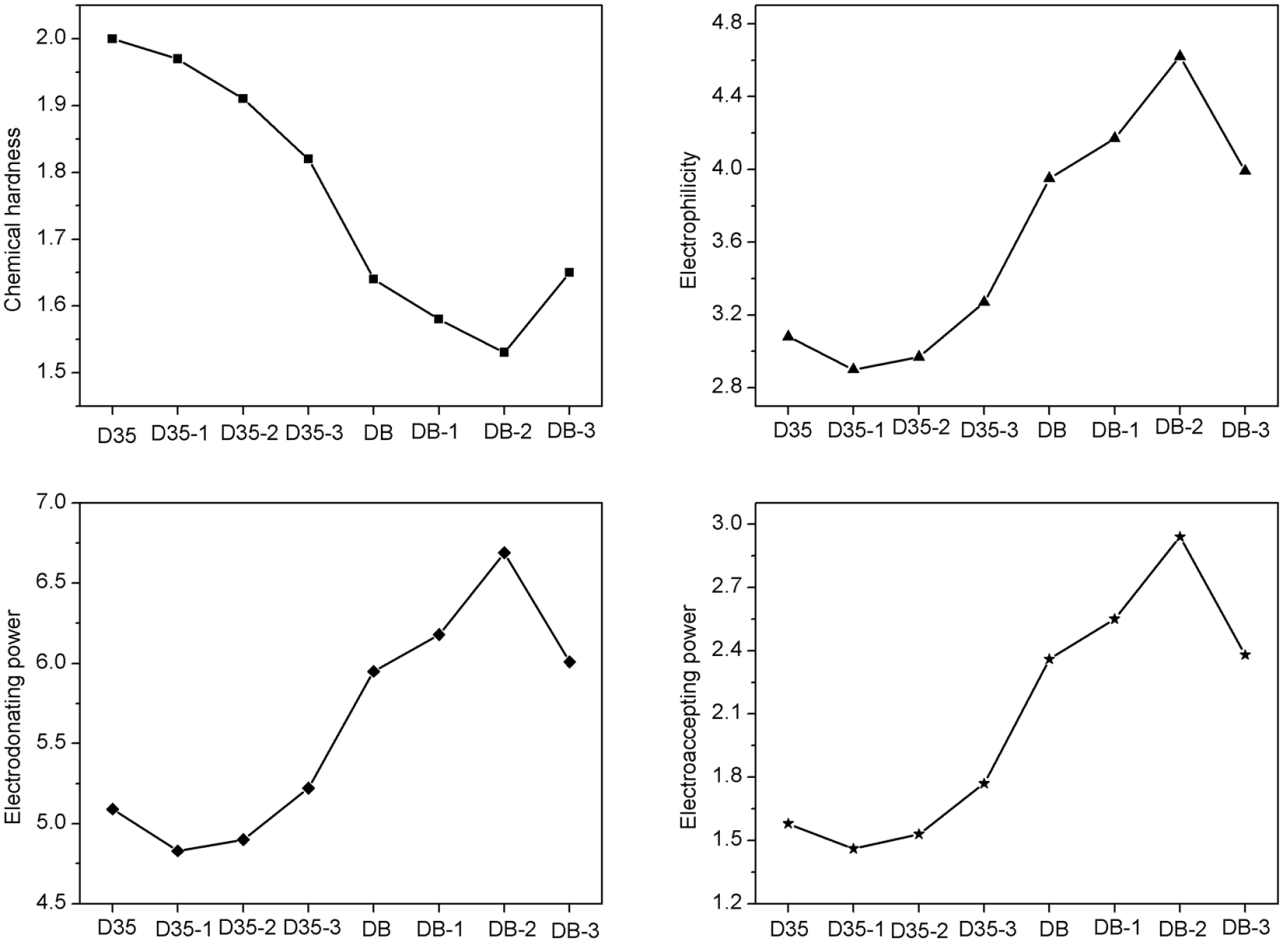
Calculated chemical reactivity parameters of the original and designed dyes.

Figure 11 shows the calculated chemical reactivity parameters of the original and designed dyes (the various parameters are listed in Table S3). As shown in Fig. 11, D35-3 has the lowest chemical hardness and highest electroaccepting power among the dye D35 and its derivatives, which would result in the greater short-circuit current density, thereby obtaining a better PCE^57^. Moreover, the dye DB-2 shows the lowest chemical hardness and highest electroaccepting power among the dye DB and its derivatives. It is surprising that DB-2 exhibits the lowest chemical hardness and highest electroaccepting power among the investigated dyes, suggesting that the dye DB-2 would have the prominent Jsc, and consequently bring the prominent PCE. With regard to electrophilicity(*ω*), the higher electrophilicity leads to the higher energetic stability by acquiring electrons from the environment^58^. It can be found from Fig. 11 that the dye D35-3 and DB-2 exhibit the greatest electrophilicity among the two series dyes, respectively, which indicates that the two dyes possess the highest energetic stability by acquiring electrons from the environment. In addition, the calculated electron-donating powers of the original and designed dyes show the same tendency as the obtained electrophilicities and electroaccepting powers, which make against improving the donating electrons ability of the dye D35-3 and DB-2^59^.

### Ground- and excited-state properties under external electric field

**Figure 12:**
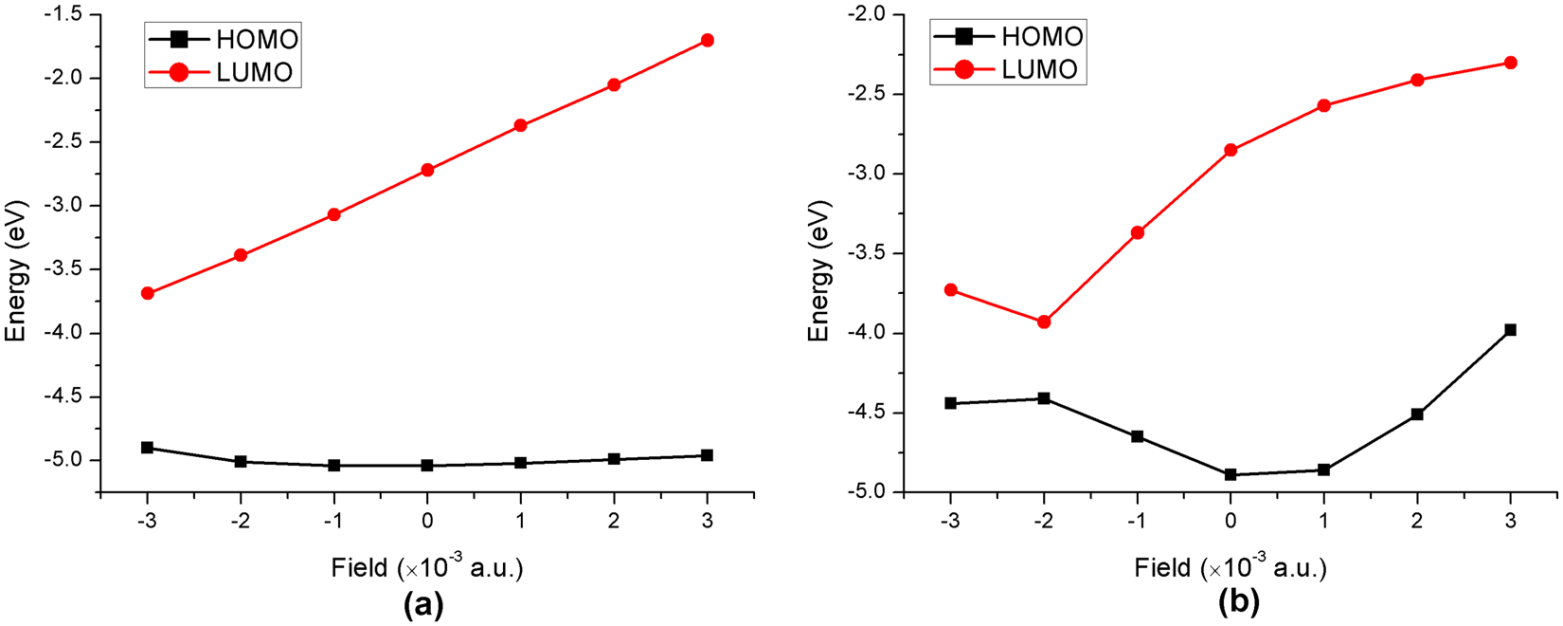
Effect of external electric field on the frontier molecular orbital energies of the dye (**a**) D35 and (**b**) DB.

The geometries of the dyes D35 and DB were optimized under the external electric field of −3.0 × 10^−3^~3.0 × 10^−3^ a.u. In this work, the direction from the electron donor to the electron acceptor was determined to be the positive direction. The trend of obtained HOMO and LUMO energies with the increasing electric field intensity is presented in Fig. 12a,b. It can be found in Fig. 12a that for the dye D35, the HOMO energies have no obvious change under the electric field of −3.0 × 10^−3^~3.0 × 10^−3^ a.u. However, the LUMO energies of D35 increase gradually with the electric field increasing from 1.0 × 10^−3^ a.u. to 3.0 × 10^−3^ a.u. resulting in gradually increasing energy gaps under the same variation tendency of electric field (see Table S4). To the contrary, the LUMO energies of D35 decrease gradually with the electric field increasing from −1.0 × 10^−3^ a.u. to −3.0 × 10^−3^ a.u. leading to the decrease of energy gap under the same variation tendency of electric field. In addition, it can be found from Fig. 12b that for the dye DB, the HOMO and LUMO energies are increased gradually along with the increased electric field from 1.0 × 10^−3^ a.u. to 3.0 × 10^−3^ a.u., and the energy gaps are decreased by degrees under the same variation tendency of electric field. Moreover, the HOMO energies first increase and then decrease when the electric field is increased from −1.0 × 10^−3^ a.u. to −3.0 × 10^−3^ a.u. and the LUMO energies first decrease and then increase under the same variation tendency of electric field, in which the energy gap reaches the minimum value under the electric field of −2.0 × 10^−3^ a.u.

**Figure 13:**
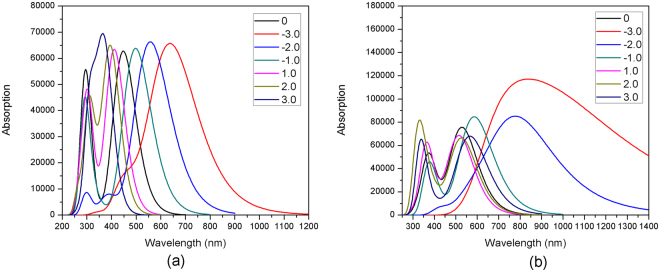
Simulated absorption spectra for the dye (**a**) D35 and (**b**) DB under the external electric field of −3.0~3.0 × 10^−3^ a.u.

Under the different external electric field, the excited state properties of the dyes were calculated in the electric field, and the spectra of the two dyes under the external electric field of −3.0 × 10^−3^~3.0 × 10^−3^ a.u. are presented in Fig. 13a,b, and Table S5 shows the corresponding excited state properties with oscillator strength *f* > 1. It can be found from Fig. 13a that for the dye D35, the maximum absorption peak is red-shifted along with the gradual increase of the electric field intensity from −1.0 × 10^−3^ to −3.0 × 10^−3^ a.u., and the red-shift values are 49.92 nm, 109.53 nm and 190.85 nm for F = −1.0 × 10^−3^, −2.0 × 10^−3^ and −3.0 × 10^−3^ a.u. respectively, compared with the maximum absorption peak (447.49 nm) in the absence of electric field. When the electric field increases from 1.0 × 10^−3^ to 3.0 × 10^−3^ a.u., the corresponding maximum absorption peaks of D35 have a blue-shift by degrees compared with the maximum absorption peak (447.49 nm) with no electric field (blue-shift of 36.60 nm, 52.58 nm and 76.17 nm for 1.0 × 10^−3^, 2.0 × 10^−3^ and 3.0 × 10^−3^ a.u., respectively). Combining with the change of energy gap in the electric field, it can be found that the decrease or increase of energy gap in the electric field would cause the red- or blue-shift of absorption peak. Moreover, as shown in Fig. 13b for the dye DB, the red-shift of maximum absorption peak occurs with the increase of electric field from 1.0 × 10^−3^ to 3.0 × 10^−3^ a.u., which corresponds to the decrease of energy gap. In addition, the maximum absorption peaks are 585.59 nm, 793.91 nm and 722.46 nm for F = −1.0 × 10^−3^, −2.0 × 10^−3^ and −3.0 × 10^−3^ a.u., and the corresponding energy gaps are 1.28 eV, 0.48 eV and 0.71 eV, respectively.

### Absorption and intermolecular charge transfer of the dimers

**Figure 14:**
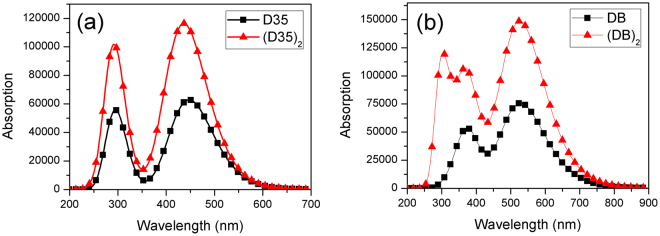
Simulated absorption spectra of the monomers and dimers for (**a**) D35 and (**b**) DB

For studying an impact of aggregation on the optical properties of dyes, absorption spectra for the dimers corresponding to the researched dyes were calculated based on the dimers geometries. The obtained absorption spectra of the dimers are presented in Fig. 14 and Fig. S4 (see Supplementary Fig. S4), and Table S6 shows the transition properties corresponding to the main absorption peaks. The absorption spectra of dimers have the same shape with that of the corresponding monomers except the dyes DB and DB-3, in which the absorption spectra of (DB)_2_ and (DB-3)_2_ present an extra absorption band in comparison with the corresponding monomers. However, noted that the peak strengths for all dimers are greater than those of the monomers. In addition, Table S6 (see Supplementary Table S6) also shows that the absorption peaks for the dimers exhibit different degrees of blue-shifts compared with that those of the monomers. By comparing with the maximum absorption peaks of the monomers, those of the dimers have the blue-shifts of 12.96 nm, 17.31 nm, 8.05 nm, 13.66 nm, 3.33 nm, 7.94 nm, 15.78 nm and 6.46 nm for (D35)_2_, (D35-1)_2_, (D35-2)_2_, (D35-3)_2_, (DB)_2_, (DB-1)_2_, (DB-2)_2_ and (DB-3)_2_, respectively.

**Figure 15:**
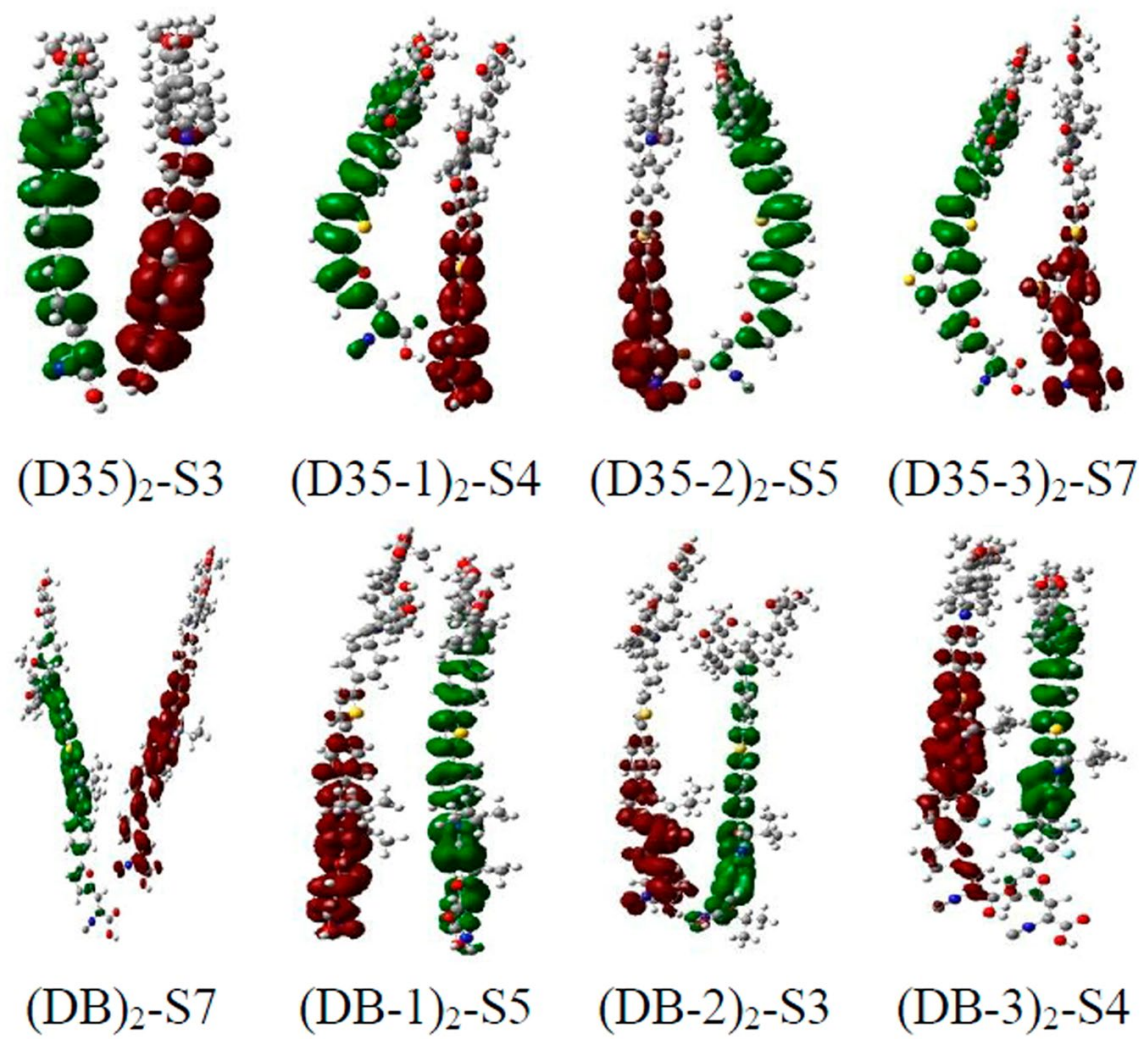
Selected charge difference density (CDD) charts for the dimers.

In order to intuitively observe the charge transfer process in the dimers under photo-excitation, the CDD analysis method was adopted, and CDD results for the first thirty excited states of the dimers are shown in Fig. S5–S12 (see Supplementary Fig. S5–S12). The selected charts corresponding to the first charge completely separated state of the dimers are presented in Fig. 15, in which the electrons and holes are distributed on the two monomers, respectively. It can be found from Fig. S5 that for the dimer (D35)_2_, the charge distribution of excited states S4, S10, S11, S20 and S23 are similar to that of the excited state S3. It is noteworthy that for the excited states S20 and S23, the holes are distributed in the donor part of a monomer and the electrons are distributed in the conjugated bridge and acceptor part of another monomer. Similarly, as shown in Fig. S6–S12, the excited states S4, S5, S11, S13, S23, S25, S26 for (D35-1)_2_, S5, S6, S14, S21, S24, S29, S30 for (D35-2)_2_, S7, S8, S12, S16, S25, S26 for (D35-3)_2_, S7, S9, S13, S14, S24, S30 for (DB)_2_, S5-S7, S11, S23-S25 for (DB-1)_2_, S3, S4, S7, S8, S18, S20, S29 for (DB-2)_2_, S4, S6, S11, S14-S16, S24, S28 for (DB-3)_2_, all belong to the charge completely separated state.

In order to investigate the intermolecular charge transfer in the dye aggregation, the lateral intermolecular charge transfer rate between dimers was calculated based on the non-adiabatic Marcus theory, in which the Gibbs free energy has no obvious change at the initial and final state due to the two identical dyes for the stacked dimers^60,61^:

10 $${\rm{\kappa }}=\frac{4{{\rm{\pi }}}^{2}}{{\rm{h}}}{|{{\rm{V}}}_{{\rm{ij}}}|}^{2}\frac{1}{\sqrt{4{{\rm{\pi }}{\rm{\lambda }}{\rm{\kappa }}}_{{\rm{B}}}{\rm{T}}}}\exp [-\frac{{\rm{\lambda }}}{4{{\rm{\kappa }}}_{{\rm{B}}}{\rm{T}}}]$$

where $h$ is the Planck’s constant, ${{\rm{\kappa }}}_{{\rm{B}}}$ represents the Boltzmann constant, ${\rm{T}}$ is the temperature, $|{{\rm{V}}}_{{\rm{ij}}}|$ and ${\rm{\lambda }}$ are the intermolecular electronic coupling and the reorganization energy, respectively. With the direct method, the value of $|{{\rm{V}}}_{{\rm{ij}}}|$ should be estimated as^62^:

11 $${{\rm{V}}}_{{\rm{ij}}}= < \,{\phi }_{{\rm{HOMO}}}^{{\rm{i}}}|{\rm{F}}|{{\rm{\phi }}}_{{\rm{HOMO}}}^{{\rm{j}}} > $$

where operator F is the Kohn-Sham-Fock matrix for the dimer, and ${{\rm{\phi }}}^{{\rm{j}}/{\rm{i}}}\,$stands for the two near molecular orbitals. Table 6 shows the values of ${{\rm{\lambda }}}_{{\rm{e}}}$, $|{{\rm{V}}}_{{\rm{ij}}}|$ and ${{\rm{\kappa }}}_{{\rm{e}}}$. The ${{\rm{\kappa }}}_{{\rm{e}}}$ for (D35)_2_ is two orders of magnitude greater than that for (DB)_2_. The greater ${{\rm{\kappa }}}_{{\rm{e}}}$ for (D35)_2_ increases the electron loss in the process of Dye → TiO_2_^61^. That is to say, the electron injection rate from the dye to TiO_2_ for D35 would be lower than that for DB, thereby weakens the photoelectrical properties of the dye D35, which is consistent with the experimental results^34^. Moreover, for the designed dyes based on the dye D35, the ${{\rm{\kappa }}}_{{\rm{e}}}$ are in the order of (D35-3)_2_ < (D35-1)_2_ < (D35-2)_2_, indicating that the dye D35-3 would show the most efficient electron injection from the dye to TiO_2_ among the three designed dyes. For the derivates of DB, the ${{\rm{\kappa }}}_{{\rm{e}}}$ follow the order of (DB-3)_2_ < (DB-2)_2_ < (DB-1)_2_, implying that DB-3 would have a more efficient electron injection compared with the other two DB derivates.

**Table 6:** Calculated the electron reorganization energies (λ_e_), intermolecular electronic couplings ($|{{\rm{V}}}_{{\rm{ij}}}|$) and lateral intermolecular electron transfer rates (κ_e_) for the dimers.

	(D35)_2_	(D35-1)_2_	(D35-2)_2_	(D35-3)_2_	(DB)_2_	(DB-1)_2_	(DB-2)_2_	(DB-3)_2_
λ_e_ (eV)	0.509	0.449	0.385	0.402	0.313	0.298	0.330	0.307
V (eV)	6.46 × 10^−3^	1.27 × 10^−2^	2.08 × 10^−2^	3.67 × 10^−3^	2.35 × 10^−4^	1.03 × 10^−2^	1.80 × 10^−3^	4.82 × 10^−3^
κ_e_ (s^−1^)	7.13 × 10^9^	5.24 × 10^10^	2.81 × 10^11^	7.30 × 10^9^	8.01 × 10^7^	1.81 × 10^11^	3.90 × 10^9^	4.86 × 10^7^

## Discussion

In this work, two series of novel dyes were designed with the multipolar structures for the dyes D35 and DB by the modification for their conjugated bridges. The ground- and excited-state properties of the original and designed dyes were investigated systematically via quantum chemistry methods. Moreover, the key parameters associated with ${{\rm{V}}}_{{\rm{OC}}}$ and ${{\rm{J}}}_{{\rm{SC}}}$ containing dipole moment (${{\rm{\mu }}}_{{\rm{normal}}}$), shift of the CB of semiconductor (${{\rm{\Delta }}E}_{{\rm{CB}}}$), light harvesting efficiency (LHE), driving force of electron injection (${{\rm{\Delta }}G}^{{\rm{inject}}}$), exciton binding energy (${E}_{{\rm{b}}}$) and chemical reactivity parameters were calculated. The effects of external electric field on the optical and electric properties of dyes were investigated. In order to investigate the intermolecular charge transfer in the dye aggregation, the lateral intermolecular charge transfer rate between dimers was calculated based on the non-adiabatic Marcus theory. From the above results, it can be concluded that D35-3 and DB-2 would exhibit the better optical and electrical properties due to their narrower energy gaps, widened optical absorption, longer excited state lifetimes, and larger transferred charge (${\rm{\Delta }}q$) among the two series of designed dyes. Furthermore, D35-3 and DB-2 possess the lower chemical hardness, higher electroaccepting power and electrophilicity among the two series of dyes, which would result in their prominent J_sc_, and consequently bring the better PCE. Considering the charge transfer process, the ability of hole transfer of the two series of dyes would be higher than that of the electron transfer arising from their lower hole reorganization energies. D35-3 and DB-3 would show the most efficient electron injection from the dye to TiO_2_ among the two series of designed dyes because of their lower intermolecular electron transfer rate (${{\rm{\kappa }}}_{{\rm{e}}}$). Therefore, DB-2 could be used as a candidate for high performance dyes in the field of DSSC, and rational molecular design can provide valuable reference for the synthesis of dyes with higher efficiency in the experiment.

## Methods

All calculations in this work were performed by using Gaussian 09 package^63^. The geometries of the investigated dyes were optimized without any constraint in the DFT framework^64,65^, with B3LYP^66–68^ functional at 6–31 G(d) basis set. Moreover, the excitation and emission characteristics of the dyes have been investigated with TD-DFT method^69,70^, with CAM-B3LYP^71^ functional at 6–31 G(d). The calculations in solvent employed the Conductor-like PCM (C-PCM) model^72^, and the solvent acetonitrile was adopted, which was used in the experiment^34^. In order to save computing time, the alkyl chains in the original dye molecules were replaced by the methyl due to the negligible influence of the alkyl chain length on the electronic structures of dyes^73^.

Moreover, the ground- and excited-state performances of the dye/TiO_2_ complexes have been obtained with the Ti(OH)_3_H_2_O cluster model reported by Peng *et al*.^74^, which has been proved the feasibility to reveal the photoelectrical properties of dyes by Ramkumar *et al*.^75^. The ground state structures of the dye/TiO_2_complexes were optimized via DFT//B3LYP/6–31 G(d) for C, H, O, N, S, F atoms and effective core potential (ECP) LANL2DZ^76–78^ and the accompanying basis set for Ti atom. Three-dimensional (3D) real space CDD analysis method was employed to intuitively display the electron transfer performances in the isolated dyes, dye/TiO_2_ complexes and dimers under the photo-excitation, which has been validated in the previous works^79–82^. The charts of density of state (DOS) and partial density of state (PDOS) for the dye/TiO_2_ complexes were displayed via the Multiwfn 3.3.7 package^83^. According to the previous literatures^84,85^, under the obtained ionization potentials (IPs) and electron affinities (EAs) of the dyes, we calculate the chemical hardness (h), electrophilicity (ω), electrodonating power (ω^−^) and electroaccepting power (ω^+^) of dyes via the following equations:

12 $${\rm{IP}}={{\rm{E}}}_{{\rm{ca}}}-{{\rm{E}}}_{{\rm{neutral}}}$$

13 $${\rm{EA}}={{\rm{E}}}_{{\rm{neutral}}}-{{\rm{E}}}_{{\rm{an}}}$$

14 $${\rm{h}}=\frac{{\rm{IP}}-{\rm{EA}}}{2}$$

15 $${{\rm{\omega }}}^{+}=\frac{{({\rm{IP}}+3{\rm{EA}})}^{2}}{16({\rm{IP}}-{\rm{EA}})}$$

16 $${{\rm{\omega }}}^{-}=\frac{{(3{\rm{IP}}+{\rm{EA}})}^{2}}{16({\rm{IP}}-{\rm{EA}})}$$

17 $${\rm{\omega }}=\frac{{({\rm{IP}}+{\rm{EA}})}^{2}}{4({\rm{IP}}-{\rm{EA}})}$$

where, ${{\rm{E}}}_{{\rm{neutral}}}$, ${{\rm{E}}}_{{\rm{ca}}}$ and ${{\rm{E}}}_{{\rm{an}}}$ represent the energies of neutral molecule, cation and anion, respectively.

## Electronic supplementary material

SUPPLEMENTARY INFO
