# Supplementary material for: Physical Insight on Mechanism of Photoinduced Charge Transfer in Multipolar Photoactive Molecules
Source: Sci Rep. 2018 Jul 4;8:10089. doi: 10.1038/s41598-018-28429-3 (PMC6031644; doi:10.1038/s41598-018-28429-3)
Supplement: Supplementary file 1 — SUPPLEMENTARY INFO [file 41598_2018_28429_MOESM1_ESM.pdf]

Supplementary Information

Physical Insight on Mechanism of Photoinduced Charge Transfer in Multipolar Photoactive Molecules

Yuanzuo Li,[*1] Chaofan Sun,[1,2] Peng Song,[3,4] Fengcai Ma,[3] Nawee Kungwan,[*5,6] and Mengtao Sun,[*4]

[1]*College of Science, Northeast Forestry University, Harbin 150040, Heilongjiang, China;*

[2]*Institute of Atomic and Molecular Physics, Jilin University, Changchun 130012, China;*

[3]*Department of Physics, Liaoning University, Shenyang 110036, China;*

[4] *School of Mathematics and Physics, Beijing Key Laboratory for Magneto-Photoelectrical Composite and Interface Science, University of Science and Technology Beijing, Beijing 100083, China;*

[5]*Department of Chemistry, Faculty of Science, Chiang Mai University, Chiang Mai 50200, Thailand;*

[6]*Center of Excellence in Materials Science and Technology, Chiang Mai University, Chiang Mai 50200, Thailand*

Corresponding authors:

E-mail: **yzli@nefu.edu.cn (Y. Z. Li), naweekung@gmail.com (N. Kungwan) and mengtaosun@ustb.edu.cn (M. T. Sun).**

Contents:

Table S1. Calculated transition properties of D35-X (X=1,2,3) /TiO_2 complex in acetonitrile.

Table S2. Calculated transition properties of DB-X (X=1,2,3) /TiO_2 complex in acetonitrile.

Table S3. Calculated chemical reactivity parameters of the original and designed dyes, containing electron affinity (*A*), ionization potential (*I*), chemical hardness (*h*), electrophilicity (ω), electrodonating power (ω^-) and electroaccepting power (ω^+)

Table S4. Calculated frontier molecular orbital energies of the dyes D35 and DB under the external electric field (eV).

Table S5. Calculated transition properties of D35 and DB under different external electric field.

Table S6. Obtained transition properties of the monomers and dimers.

Figure S1. Diagrams of total and partial density of state for dye/TiO_2 complexes in acetonitrile.

Figure S2. Simulated absorption spectra of isolated dyes and dye/TiO_2 complexes in acetonitrile.

Figure S3. Charge difference density (CDD) charts for the first twenty excited states of D35/TiO_2 complex.

Figure S4. Simulated absorption spectra of the monomers and dimers.

Figure S5. Charge difference density (CDD) charts for the first thirty excited states of $(D35)_2$.

Figure S6. Charge difference density (CDD) charts for the first thirty excited states of $(D35\text{-}1)_2$.

Figure S7. Charge difference density (CDD) charts for the first thirty excited states of $(D35\text{-}2)_2$.

Figure S8. Charge difference density (CDD) charts for the first thirty excited states of $(D35\text{-}3)_2$.

Figure S9. Charge difference density (CDD) charts for the first thirty excited states of $(DB)_2$.

Figure S10. Charge difference density (CDD) charts for the first thirty excited states of $(DB\text{-}1)_2$.

Figure S11. Charge difference density (CDD) charts for the first thirty excited states of $(DB\text{-}2)_2$.

Figure S12. Charge difference density (CDD) charts for the first thirty excited states of $(DB\text{-}3)_2$.

Table S1. Calculated transition properties of D35-X (X=1, 2, 3) /TiO_2 complex in acetonitrile

State	*E* (eV)	λ_{abs} (nm)	Contribution MO	Strength *f*
D35/TiO_2				
S1	2.6840	461.94	(0.60922)H→L	1.6968
S2	3.7638	329.41	(0.48156)H-2→L	0.0732
S3	4.1521	298.60	(0.63755)H→L+5	1.0029
S4	4.2747	290.04	(0.50927)H→L+4	0.2127
S5	4.3686	283.81	(0.37766)H→L+7	0.1099
S6	4.4740	277.12	(0.40809)H-10→L	0.0896
S7	4.5612	271.82	(0.66550)H-1→L	0.0034
S8	4.6491	266.69	(0.55300)H→L+1	0.0637
S9	4.6994	263.83	(0.62165)H→L+2	0.0014
S10	4.7416	261.48	(0.36529)H→L+13	0.0136
D35-1/TiO_2				
S1	2.7019	458.87	(0.53659)H→L	1.8019
S2	3.5950	344.88	(0.46446)H-1→L	0.3566
S3	3.9834	311.25	(0.42402)H→L+4	0.3601
S4	4.0856	303.47	(0.61906)H→L+5	1.0099
S5	4.3235	286.77	(0.52867)H→L+10	0.0799
S6	4.4360	279.50	(0.35796)H-1→L+1	0.0632
S7	4.5300	273.69	(0.55385)H→L+2	0.0006
S8	4.5542	272.24	(0.37992)H-1→L+4	0.0285
S9	4.6401	267.20	(0.56250)H→L+3	0.0001
S10	4.7054	263.49	(0.37063)H-3→L	0.0144
D35-2/TiO_2				
S1	2.8784	430.74	(0.46572)H-1→L	2.3658
S2	3.4906	355.19	(0.34811)H→L+4	0.4663
S3	3.9453	314.26	(0.51872)H→L	0.2878
S4	4.0690	304.70	(0.62221)H→L+5	1.0344
S5	4.2008	295.15	(0.32167)H→L+6	0.0257
S6	4.3225	286.83	(0.62415)H→L+10	0.0397
S7	4.5737	271.08	(0.30427)H-3→L	0.0082
S8	4.6376	267.35	(0.29415)H-11→L	0.0064
S9	4.6762	265.14	(0.31465)H-1→L+4	0.0101
S10	4.6951	264.07	(0.46224)H→L+2	0.0001
D35-3/TiO_2				
S1	2.6193	473.34	(0.48220)H-1→L	1.7930
S2	3.3961	365.08	(0.34582)H-1→L	0.4981
S3	3.6402	340.60	(0.43184)H-1→L+1	0.0957
S4	3.8859	319.06	(0.40318)H→L+5	0.6266
S5	4.0713	304.53	(0.58023)H→L+6	0.8836
S6	4.0838	303.60	(0.42278)H-4→L	0.2194

S7	4.2829	289.49	(0.37559)H-3→L	0.0142
S8	4.3197	287.02	(0.57445)H→L+10	0.0408
S9	4.4536	278.39	(0.48301)H-10→L	0.0673
S10	4.5089	274.98	(0.31038)H-1→L+5	0.0156

Table S2. Calculated transition properties of DB-X (X=1, 2, 3) /TiO_2 complex in acetonitrile.

State	*E* (eV)	λ_{abs} (nm)	Contribution MO	Strength *f*
DB/TiO_2				
S1	2.3203	534.35	(0.53812)H→L	1.9103
S2	3.0882	401.48	(0.37862)H-1→L	0.2226
S3	3.2411	382.54	(0.37754)H-1→L+1	1.0609
S4	3.5478	349.47	(0.57564)H-8→L	0.0093
S5	3.6180	342.69	(0.36749)H→L+1	0.2918
S6	4.0163	308.71	(0.31702)H→L+5	0.0664
S7	4.0576	305.56	(0.31744)H-15→L	0.0417
S8	4.1174	301.12	(0.53977)H→L+6	0.9092
S9	4.1219	300.79	(0.33042)H-5→L	0.1284
S10	4.1626	297.85	(0.33711)H-1→L+5	0.0739
DB-1/TiO_2				
S1	2.2824	543.22	(0.60089)H-1→L	1.3952
S2	3.0704	403.81	(0.42629)H→L	0.6255
S3	3.4411	360.31	(0.49632)H-1→L+1	0.2723
S4	3.4797	356.31	(0.60243)H-9→L	0.0343
S5	3.6365	340.95	(0.35083)H→L+5	1.1184
S6	3.8018	326.12	(0.53013)H-5→L	0.1183
S7	3.8248	324.16	(0.35282)H-16→L	0.1411
S8	3.8835	319.26	(0.38776)H-2→L	0.0261
S9	4.0773	304.09	(0.61121)H→L+6	1.0624
S10	4.2850	289.34	(0.33453)H-1→L+5	0.0320
DB-2/TiO_2				
S1	2.1036	589.40	(0.60382)H-1→L	1.6226
S2	2.9256	423.79	(0.44444)H→L	0.6401
S3	3.3478	370.35	(0.67573)H-10→L	0.0121
S4	3.4045	364.18	(0.42145)H→L	0.4473
S5	3.5152	352.70	(0.33363)H→L+1	0.5249
S6	3.6994	335.14	(0.47651)H-1→L+1	0.1011
S7	3.8590	321.28	(0.52582)H-15→L	0.0031
S8	4.0742	304.32	(0.60024)H→L+6	1.0770
S9	4.1529	298.55	(0.34489)H-5→L	0.0352
S10	4.2286	293.21	(0.25956)H-2→L	0.1622
DB-3/TiO_2				
S1	2.3537	526.76	(0.52610)H→L	1.8537
S2	3.1236	396.92	(0.44415)H-1→L	0.0588
S3	3.2710	379.04	(0.37092)H-1→L+1	1.2683
S4	3.5418	350.06	(0.58757)H-8→L	0.0046
S5	3.6362	340.97	(0.36560)H→L+1	0.2942
S6	4.0481	306.28	(0.48857)H-15→L	0.0299

S7	4.0736	304.36	(0.35619)H→L+5	0.1413
S8	4.1065	301.92	(0.34845)H-5→L	0.0298
S9	4.1235	300.67	(0.56969)H→L+6	0.9992
S10	4.1776	296.78	(0.35650)H→L+1	0.0281

Table S3. Calculated chemical reactivity parameters of the original and designed dyes, containing electron affinity (*A*), ionization potential (*I*), chemical hardness (*h*), electrophilicity (ω), electrodonating power (ω^-) and electroaccepting power (ω^+)

	I (eV)	A (eV)	h (eV)	ω (eV)	ω^- (eV)	ω^+ (eV)
D35	5.51	1.51	2.00	3.08	5.09	1.58
D35-1	5.34	1.41	1.97	2.90	4.83	1.46
D35-2	5.28	1.46	1.91	2.97	4.90	1.53
D35-3	5.26	1.63	1.82	3.27	5.22	1.77
DB	5.23	1.96	1.64	3.95	5.95	2.36
DB-1	5.21	2.05	1.58	4.17	6.18	2.55
DB-2	5.28	2.23	1.53	4.62	6.69	2.94
DB-3	5.28	1.98	1.65	3.99	6.01	2.38

Table S4. Calculated frontier molecular orbital energies of the dyes D35 and DB under the external electric field (eV)

	F×10^{-3} (a.u.)	H	L	Δ_{H-L}
D35	0	-5.04	-2.72	2.32
	1.0	-5.02	-2.37	2.65
	2.0	-4.99	-2.05	2.94
	3.0	-4.96	-1.70	3.26
	-1.0	-5.04	-3.07	1.97
	-2.0	-5.01	-3.39	1.62
	-3.0	-4.90	-3.69	1.21
DB	0	-4.89	-2.85	2.04
	1.0	-4.86	-2.57	2.29
	2.0	-4.51	-2.41	2.10
	3.0	-3.98	-2.30	1.68
	-1.0	-4.65	-3.37	1.28
	-2.0	-4.41	-3.93	0.48
	-3.0	-4.44	-3.73	0.71

Table S5. Calculated transition properties of D35 and DB under different external electric field

Dye	F^a ($\times 10^{-3}$ a.u.)	State	λ_{abs} (nm/eV)	Contribution MO	Strength f
D35	1.0	S1	410.89/3.0174	(0.60039)H→L	1.5656
		S3	304.97/4.0654	(0.63259)H→L+1	1.0654
	2.0	S1	394.91/3.1395	(0.61961)H→L	1.5936
		S2	311.29/3.9829	(0.60868)H→L+1	1.0625
	3.0	S1	371.32/3.3390	(0.64337)H→L	1.5685
		S2	314.71/3.9396	(0.54452)H→L+1	1.1319
	-1.0	S1	497.41/2.4926	(0.61052)H→L	1.5760
	-2.0	S1	557.02/2.2258	(0.59018)H→L	1.6375
	-3.0	S1	638.34/1.9423	(0.59771)H→L	1.6106
DB	1.0	S1	515.23/2.4064	(0.66742)H→L	1.6871
		S3	359.53/3.4485	(0.41773)H-2→L	1.0323
	2.0	S1	526.97/2.3528	(0.59821)H→L	1.6306
		S6	314.00/3.9485	(0.53730)H-2→L+1	1.0034
	3.0	S1	566.50/2.1886	(0.55399)H→L	1.6693
		S6	334.71/3.7043	(0.33050)H-2→L	1.2379
	-1.0	S1	585.59/2.1173	(0.41243)H→L	2.0886
		S4	375.49/3.3019	(0.40435)H-6→L	1.0501
	-2.0	S1	793.91/1.5617	(0.52734)H→L	1.9817
	-3.0	S3	722.46/1.7161	(0.56240)H→L+1	1.7043

[a] represents the external electric field

Table S6. Obtained transition properties of the monomers and dimers

Dye	State	*E* (eV)	λ_{abs} (nm)	Contribution MO	Strength *f*
D35	S1	2.7706	447.49	(0.61188)H→L	1.5501
	S3	4.1499	298.76	(0.64108)H→L+2	1.0020
$(D35)_2$	S2	2.8533	434.53	(0.52322)H-1→L+1	2.6856
	S8	4.1760	296.90	(0.49613)H-1→L+5	1.5617
D35-1	S1	2.7760	446.63	(0.54737)H→L	1.7466
	S4	4.0927	302.94	(0.61580)H→L+2	1.0280
$(D35\text{-}1)_2$	S2	2.8879	429.32	(0.46649)H→L+1	2.6332
	S10	4.1393	299.53	(0.47664)H-1→L+4	1.6509
D35-2	S1	2.9496	420.34	(0.46287)H-1→L	2.3351
	S4	4.0757	304.21	(0.62009)H→L+2	1.0291
$(D35\text{-}2)_2$	S2	3.0072	412.29	(0.37797)H-2→L+1	4.2418
	S10	4.1108	301.60	(0.45805)H-1→L+4	1.7943
D35-3	S1	2.6705	464.27	(0.47786)H-1→L	1.7158
	S5	4.0795	303.92	(0.59347)H→L+3	0.9879
$(D35\text{-}3)_2$	S2	2.7515	450.61	(0.41086)H-2→L+1	3.0555
	S13	4.1117	301.54	(0.47108)H-1→L+6	1.6713
DB	S1	2.3443	528.88	(0.55542)H→L	1.8625
	S3	3.2921	376.62	(0.36687)H-1→L+1	1.1391
$(DB)_2$	S2	2.3592	525.55	(0.44173)H→L+1	3.1413
	S6	3.3149	374.02	(0.29041)H-3→L+3	2.0096
	S21	4.1161	301.22	(0.51944)H→L+6	1.2759
DB-1	S1	2.3070	537.43	(0.60799)H-1→L	1.3544
	S5	3.6655	338.24	(0.39180)H→L+2	1.0237
$(DB\text{-}1)_2$	S2	2.3416	529.49	(0.43314)H-3→L	2.6480
	S14	3.6802	336.89	(0.23997)H-1→L+5	2.0326
DB-2	S1	2.0807	595.89	(0.59792)H-1→L	1.5957
	S5	3.5051	353.72	(0.38934)H→L+1	0.6189
$(DB\text{-}2)_2$	S2	2.1372	580.11	(0.42423)H-2→L+1	2.9533
	S14	3.5099	353.24	(0.2887)H→L+3	1.1541
	S22	4.0863	303.42	(0.41667)H-1→L+6	1.9710
DB-3	S1	2.3635	524.58	(0.53881)H→L	1.8272
	S3	3.3154	373.97	(0.36254)H-1→L+1	1.1684
$(DB\text{-}3)_2$	S2	2.3930	518.12	(0.42583)H-1→L+1	3.4830
	S8	3.3513	369.96	(0.26352)H-3→L+3	2.1105
	S25	4.1533	298.52	(0.31098)H-1→L+8	1.4681

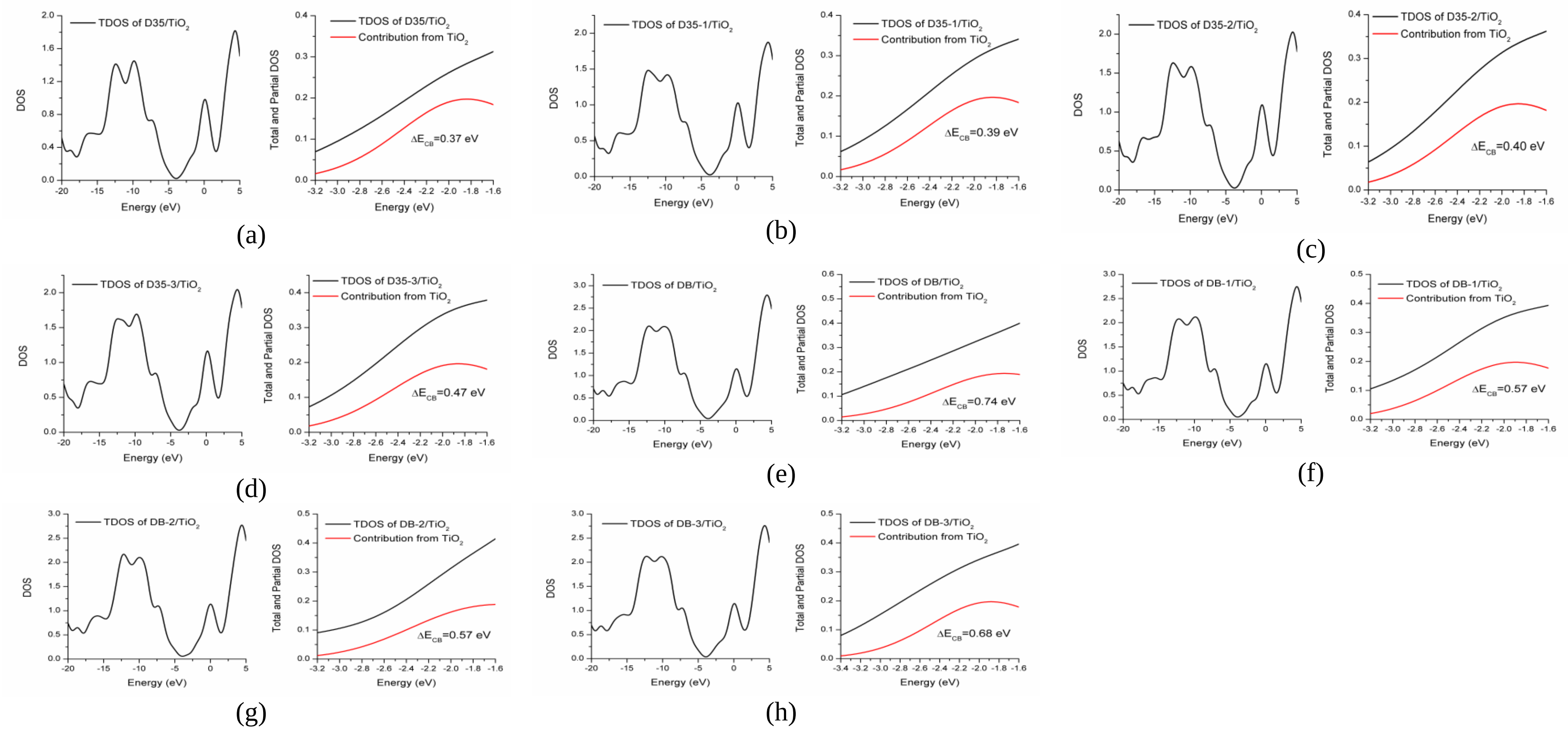

Figure S1. Diagrams of total and partial density of state for dye/TiO_2 complexes in acetonitrile.

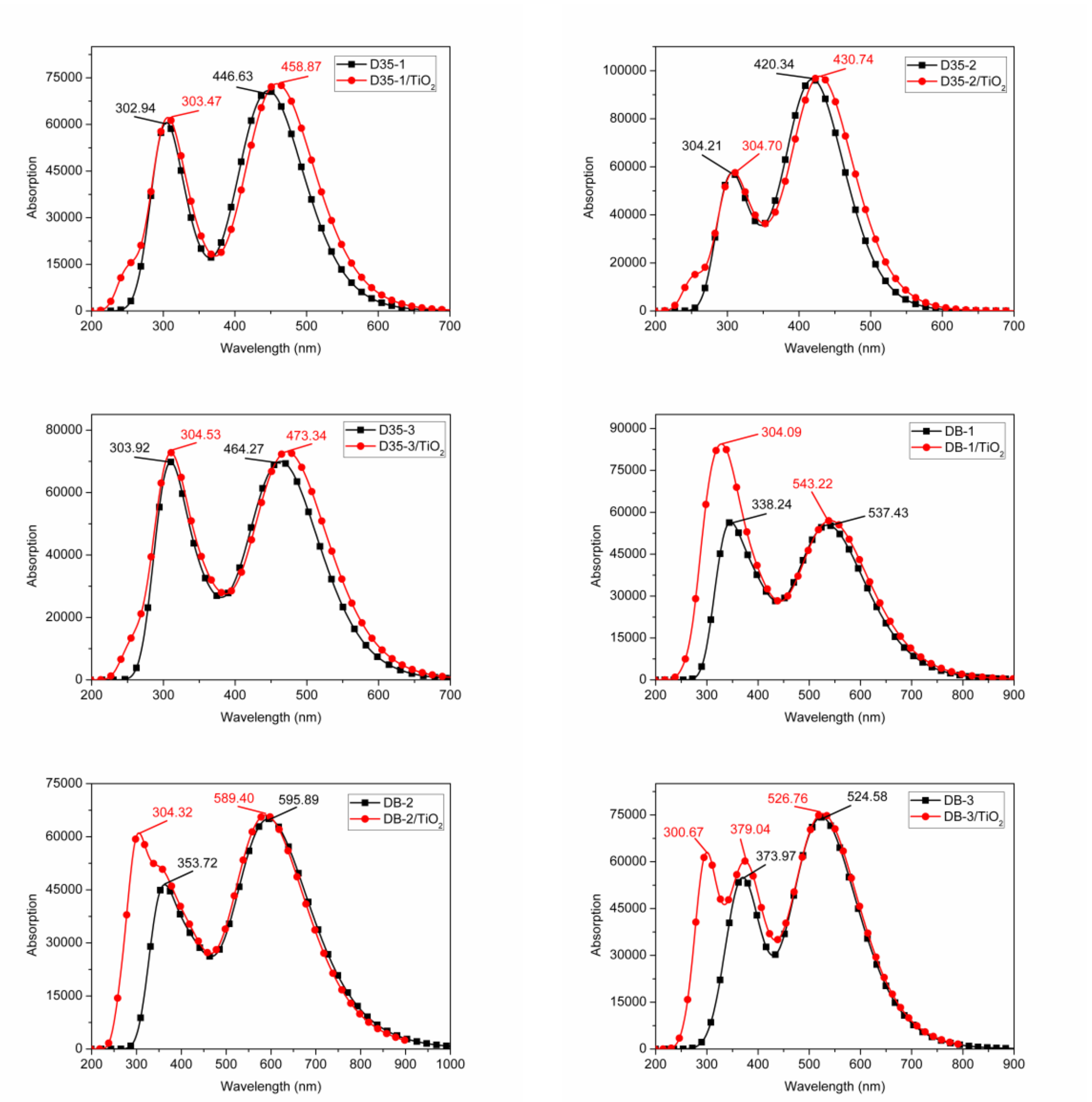

Figure S2. Simulated absorption spectra of isolated dyes and dye/TiO_2 complexes in acetonitrile.

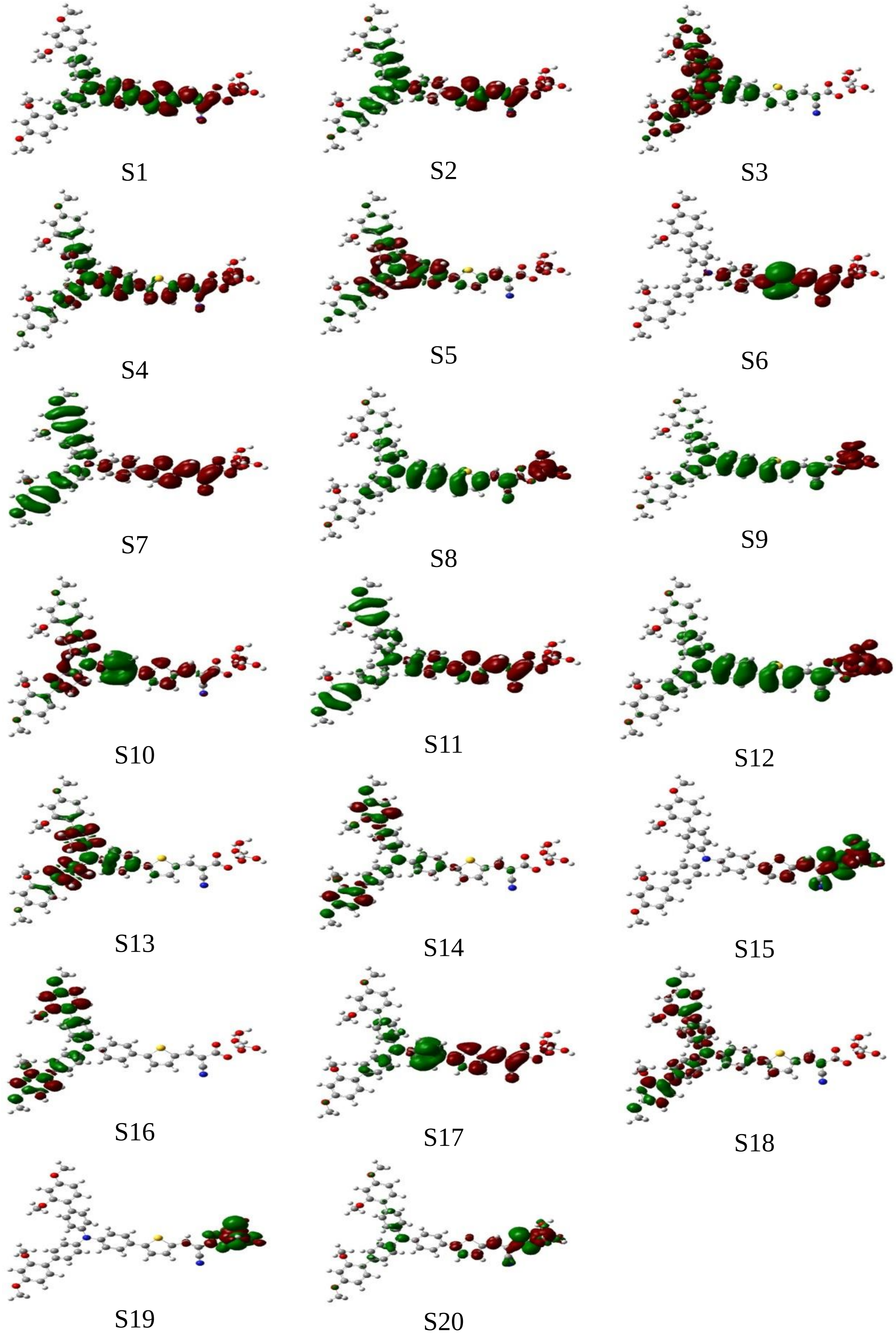

Figure S3. Charge difference density (CDD) charts for the first twenty excited states of D35/TiO_2 complex

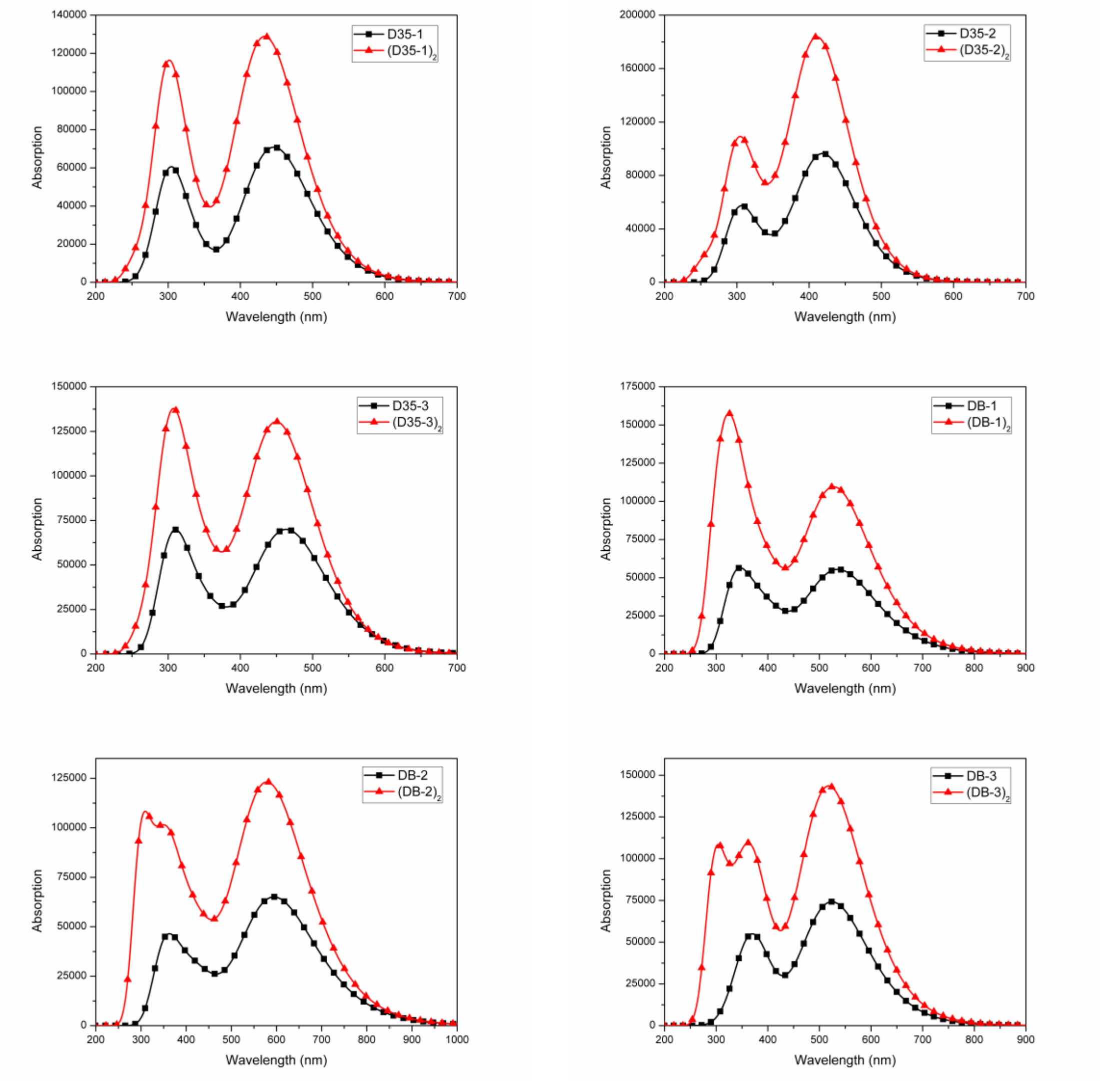

Figure S4. Simulated absorption spectra of the monomers and dimers.

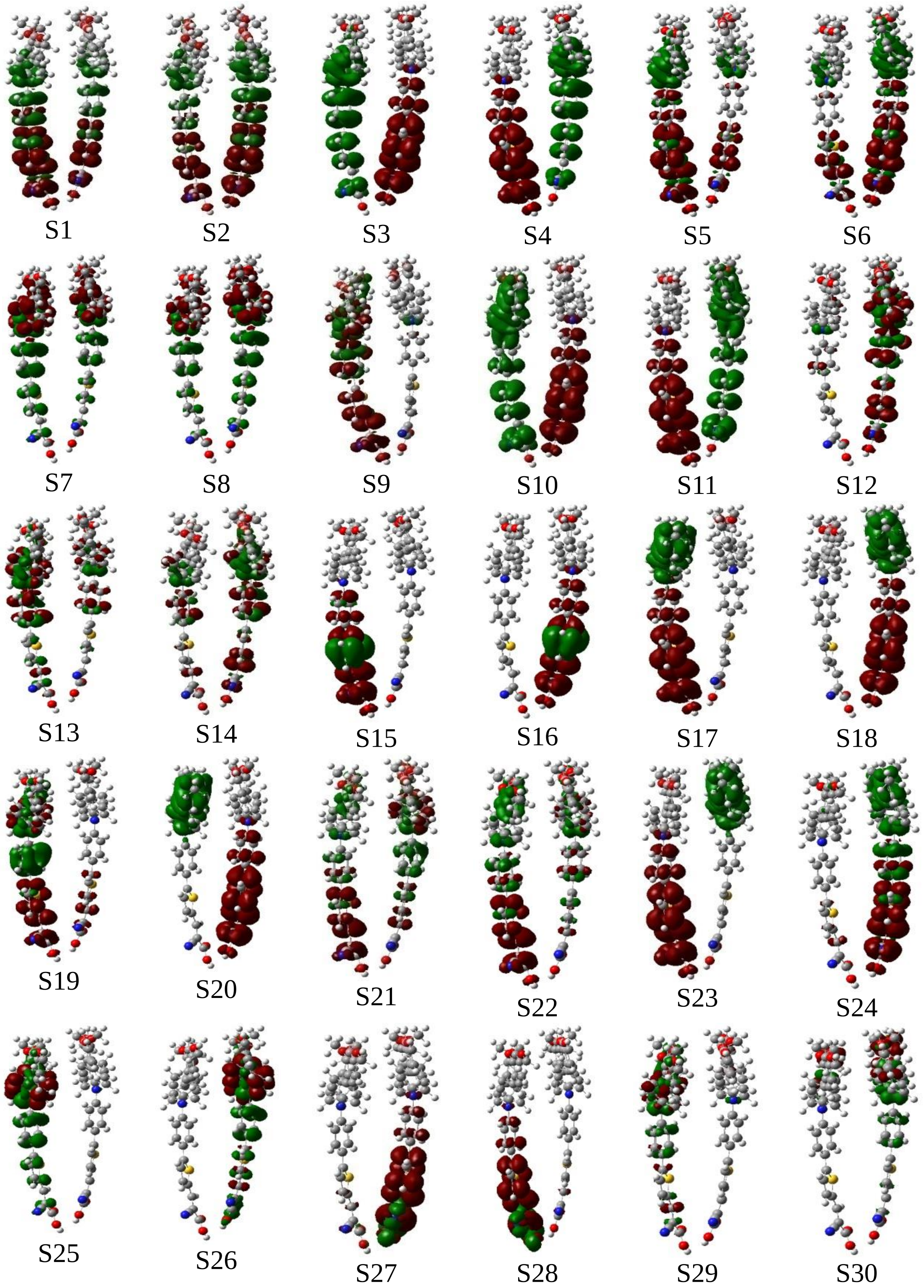

Figure S5. Charge difference density (CDD) charts for the first thirty excited states of $(D35)_2$.

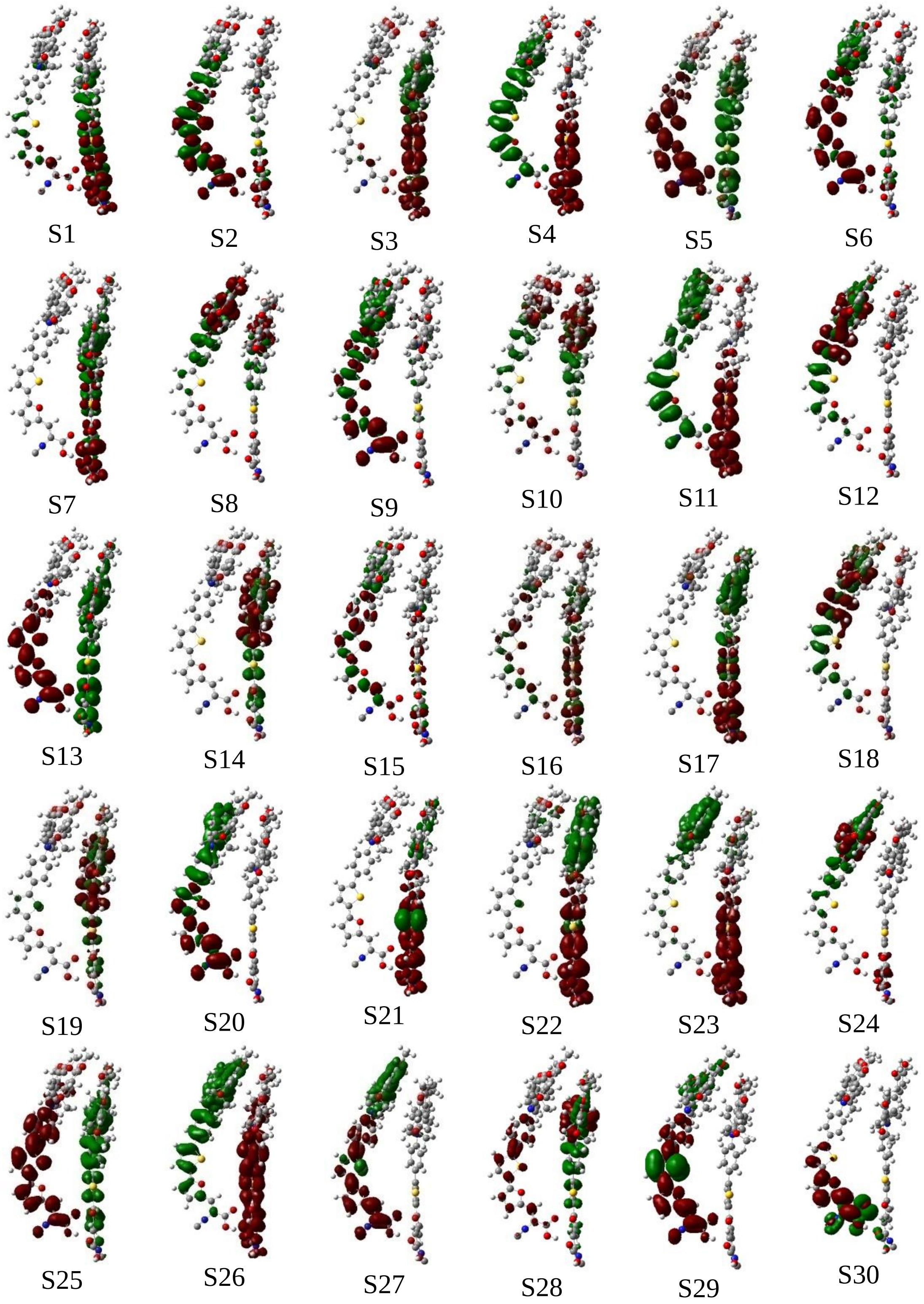

Figure S6. Charge difference density (CDD) charts for the first thirty excited states of $(D35\text{-}1)_2$.

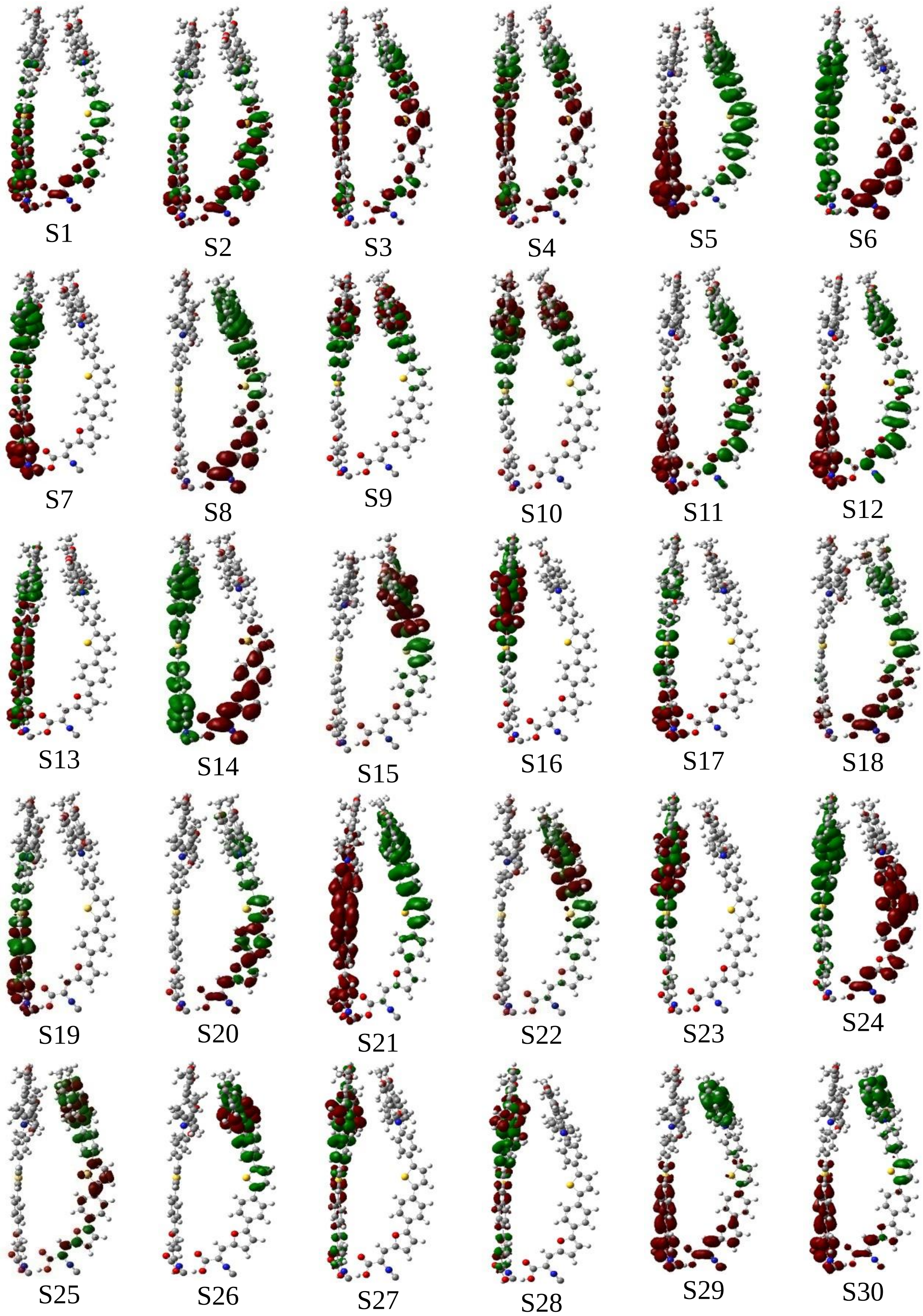

Figure S7. Charge difference density (CDD) charts for the first thirty excited states of $(D35\text{-}2)_2$.

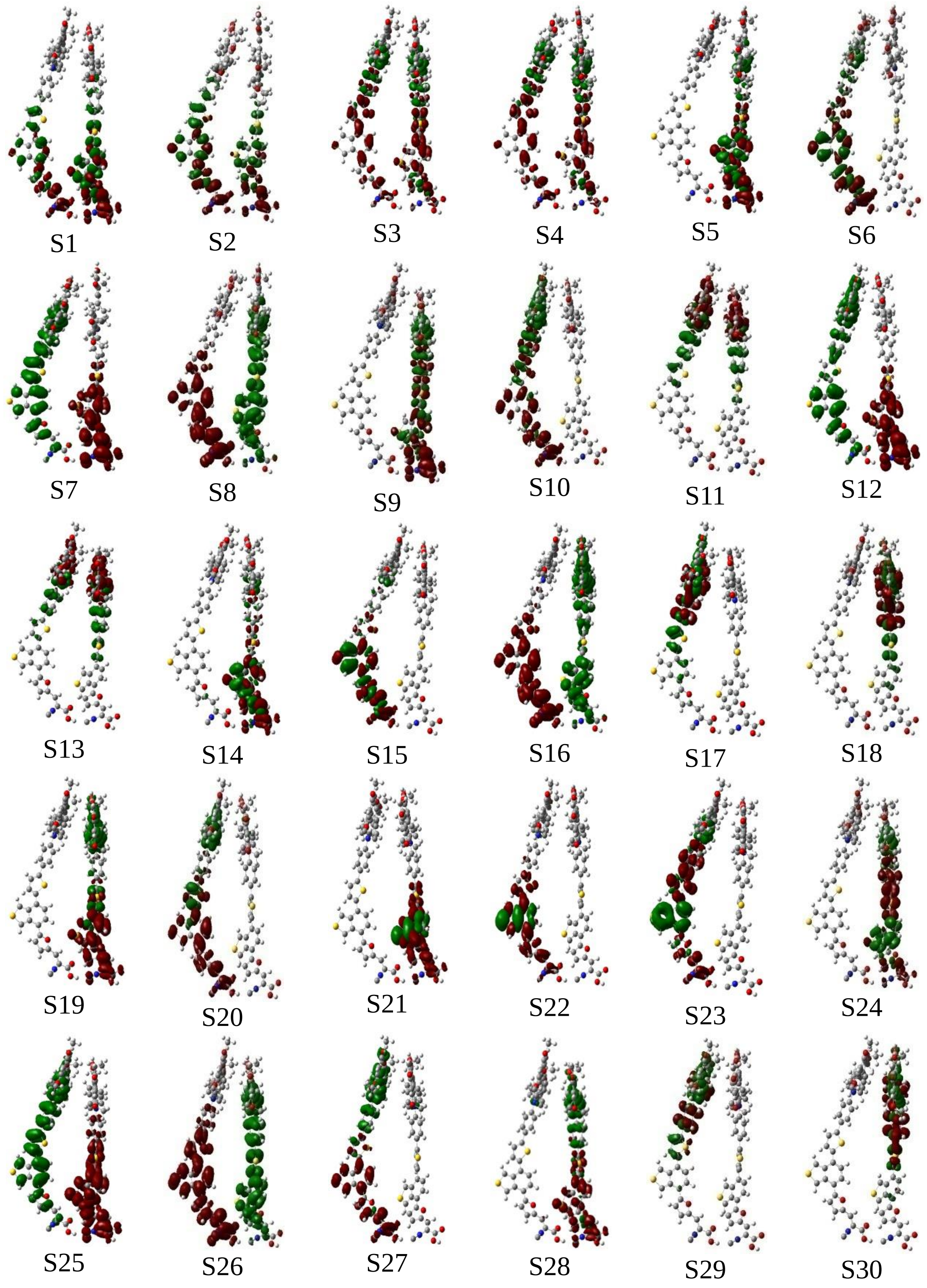

Figure S8. Charge difference density (CDD) charts for the first thirty excited states of $(D35\text{-}3)_2$.

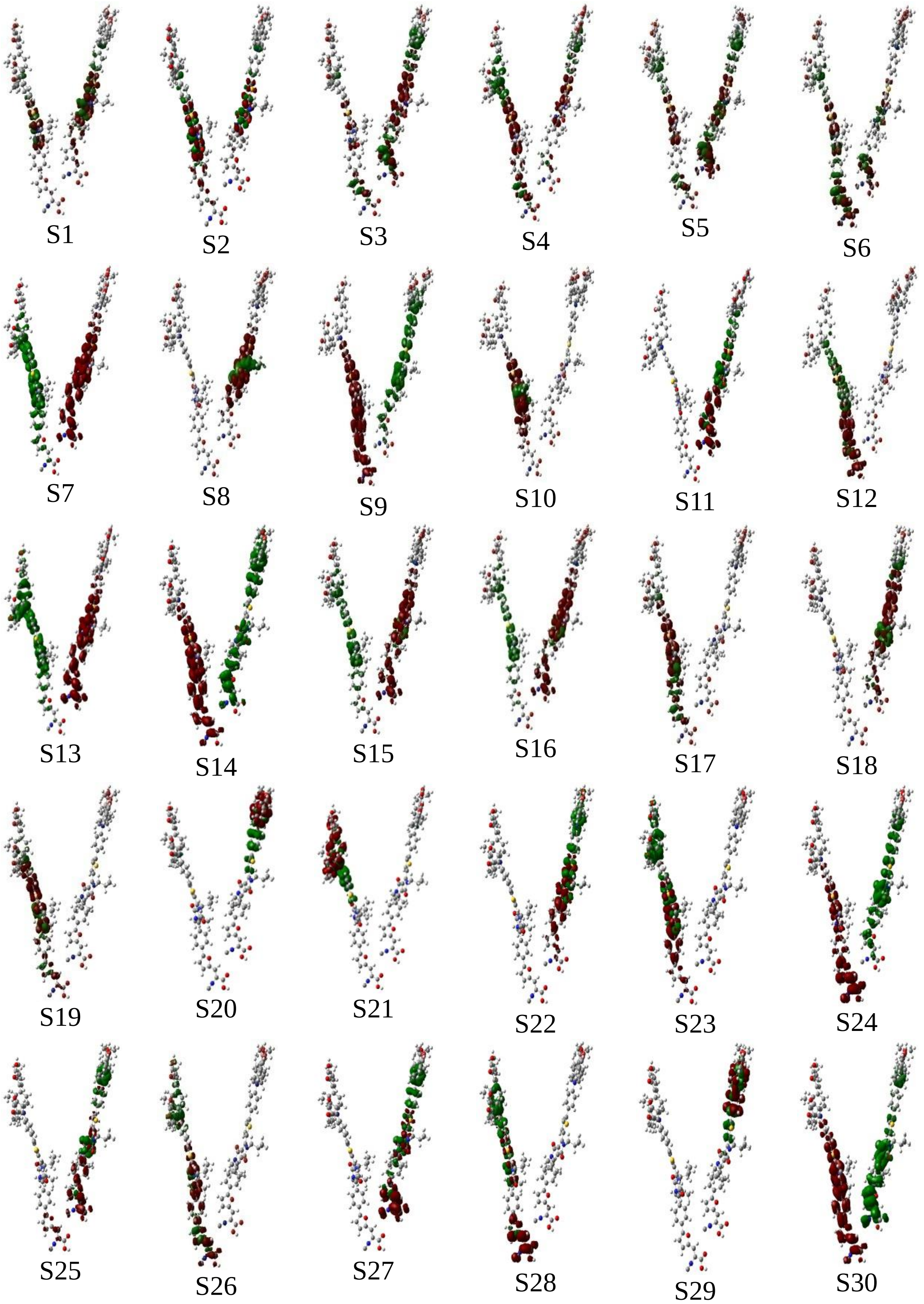

Figure S9. Charge difference density (CDD) charts for the first thirty excited states of $(DB)_2$.

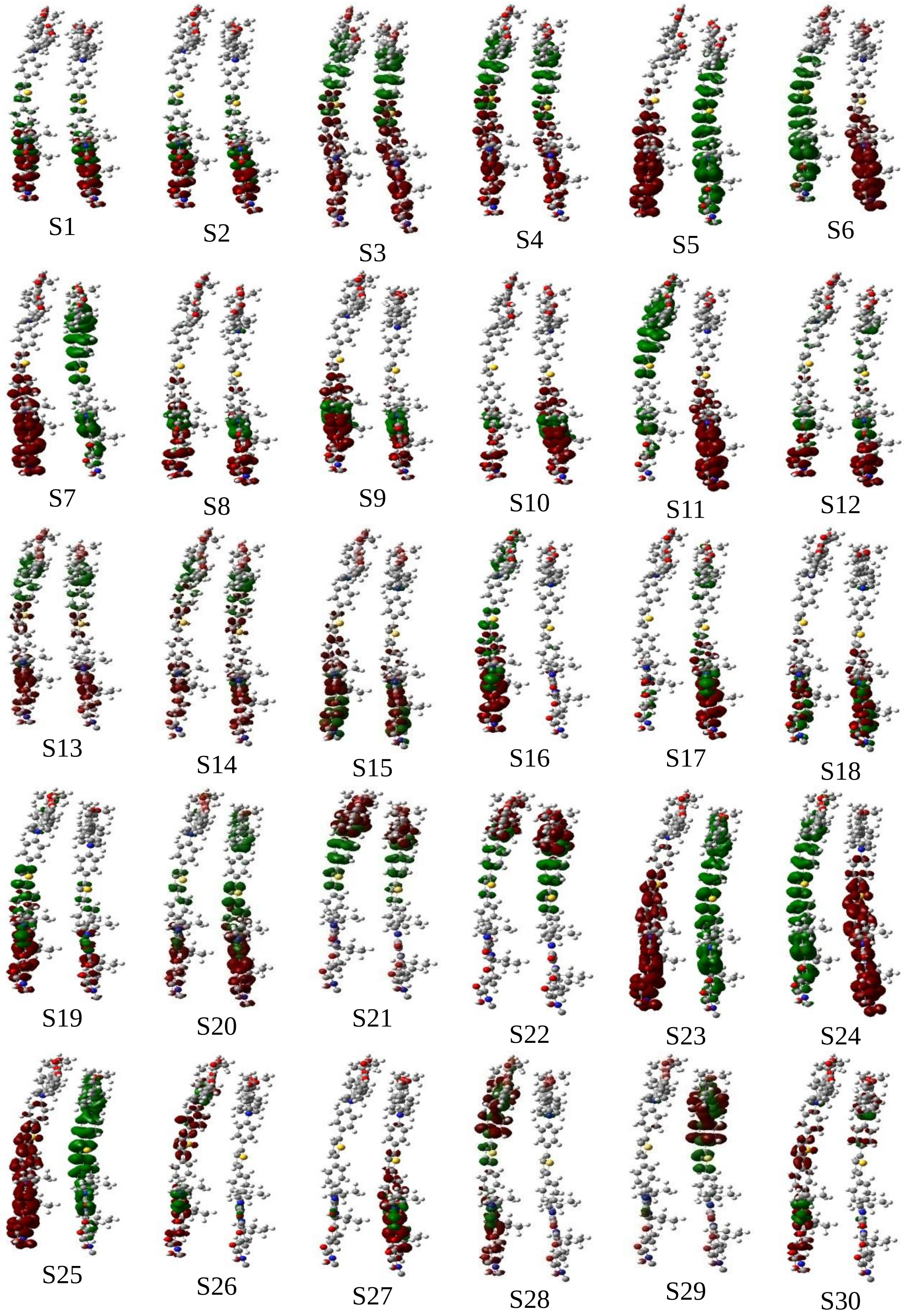

Figure S10. Charge difference density (CDD) charts for the first thirty excited states of $(DB\text{-}1)_2$.

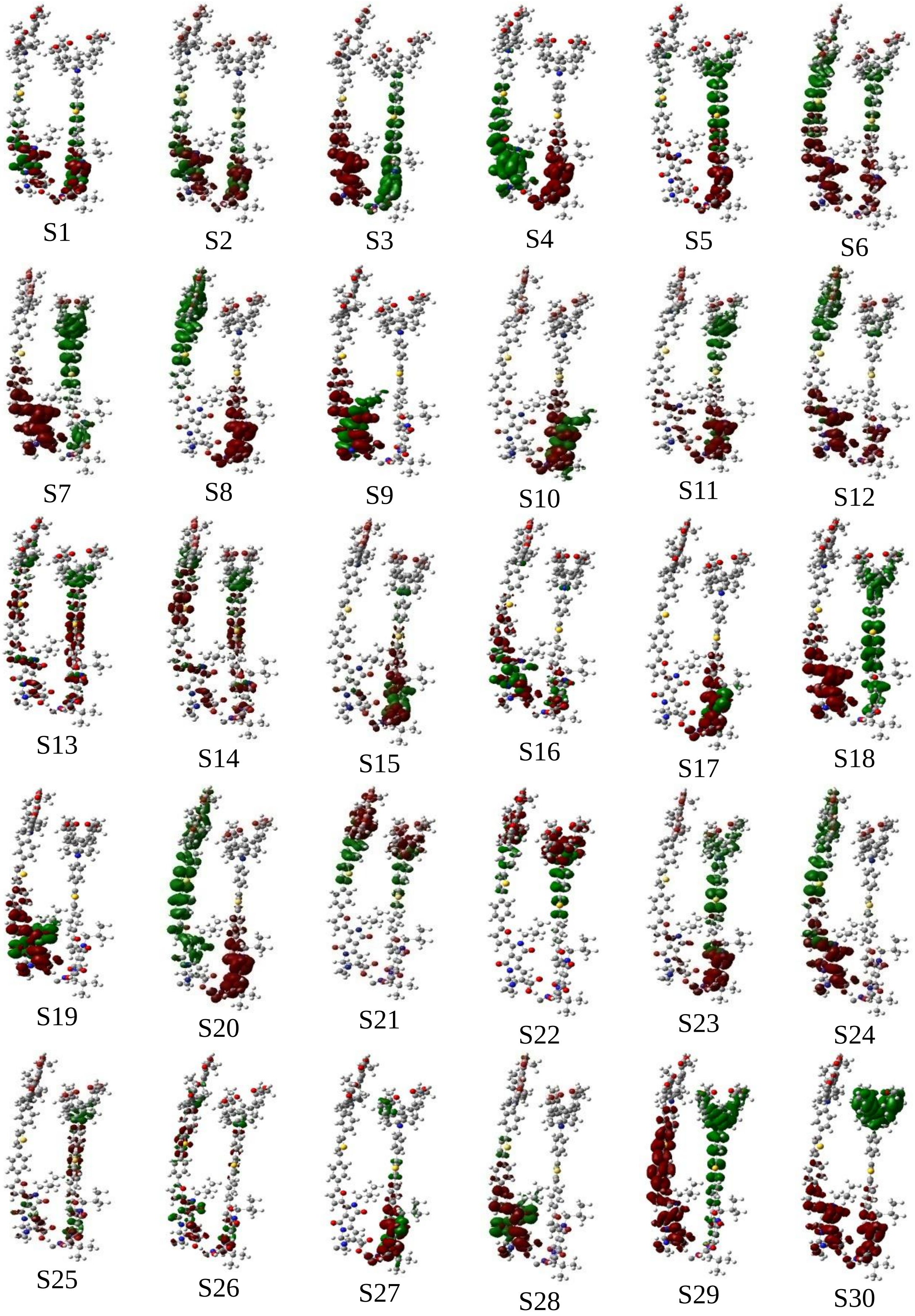

Figure S11. Charge difference density (CDD) charts for the first thirty excited states of $(DB\text{-}2)_2$.

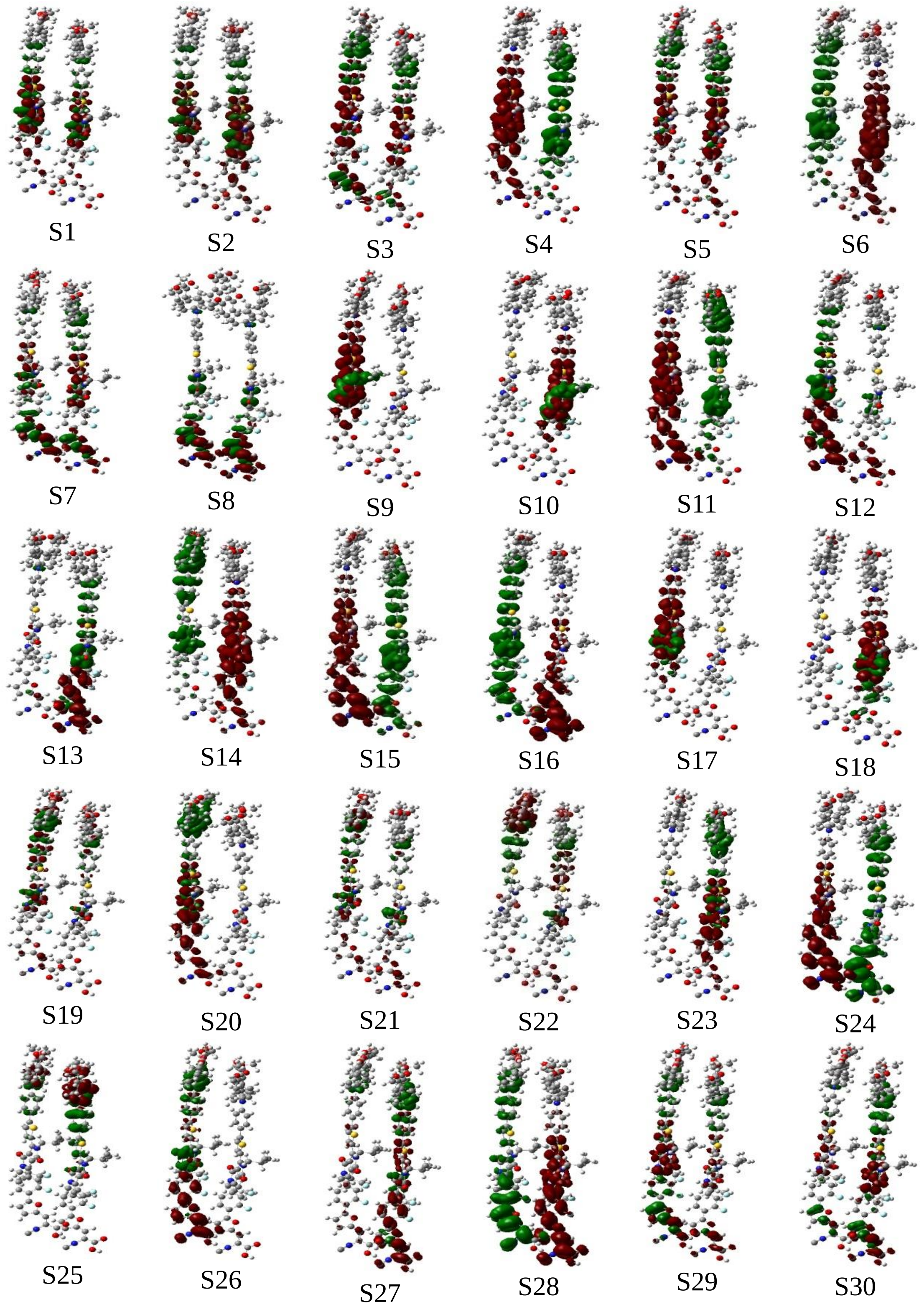

Figure S12. Charge difference density (CDD) charts for the first thirty excited states of $(DB\text{-}3)_2$.
